# Ubiquitin-conjugating enzyme complex Uev1A-Ubc13 promotes breast cancer metastasis through nuclear factor-кB mediated matrix metalloproteinase-1 gene regulation

**DOI:** 10.1186/bcr3692

**Published:** 2014-07-14

**Authors:** Zhaojia Wu, Siqi Shen, Zhiling Zhang, Weiwei Zhang, Wei Xiao

**Affiliations:** 1 College of Life Sciences, Capital Normal University, Beijing, China 100048; 2 Department of Microbiology and Immunology, University of Saskatchewan, Saskatoon, SK S7N 5E5, Canada

## Abstract

**Introduction:**

UEV1A encodes a ubiquitin-conjugating enzyme variant (Ubc13), which is required for Ubc13-catalyzed Lys63-linked polyubiquitination of target proteins and nuclear factor κB (NF-кB) activation. Previous reports have correlated the level of UEV1A expression with tumorigenesis; however, the detailed molecular events leading to tumors particularly breast cancer and metastasis are unclear. This study is to investigate roles of different UEV1 splicing variants, and its close homolog MMS2, in promoting tumorigenesis and metastasis in breast cancer cells.

**Methods:**

We experimentally manipulated the UEV1 and MMS2 levels in MDA-MB-231 breast cancer cells and monitored their effects on cell invasion and migration, as well as tumor formation and metastasis in xenograft mice. The underlying molecular mechanisms leading to metastasis were also examined.

**Results:**

It was found that overexpression of UEV1A alone, but not UEV1C or MMS2, is sufficient to induce cell invasion *in vitro* and metastasis *in vivo*. This process is mediated by NF-κB activation and requires functional Ubc13. Our experimental data establish that among NF-κB target genes, UEV1A-regulated matrix metalloproteinase-1 (MMP1) expression plays a critical role in cell invasion and metastasis. Interestingly, experimental depletion of UEV1 in MDA-MB-231 cells reduces MMP1 expression and prevents tumor formation and metastasis in a xenograft mouse model, while overexpression of MMP1 overrides the metastasis effects in UEV1-depleted cells.

**Conclusions:**

These results identify UEV1A as a potential therapeutic target in the treatment of metastasic breast cancers.

## Introduction

*UEV1*, also known as *CROC1*[1] or *CIR1*[2], encodes a ubiquitin (Ub)-conjugating enzyme variant (Uev) [3]. It was also identified as a mammalian homolog of yeast *MMS2*[4] and as a co-factor of Ubc13 [5]. Indeed, a Uev (Uev1A or Mms2) is absolutely required for Ubc13-mediated Lys(K)63-linked polyubiquitin chain assembly [5-9]. Despite their shared biochemical activity, Mms2 and Uev1A appear to function differently in mammalian cells: Ubc13-Mms2 is required for DNA-damage responses whereas Ubc13-Uev1A is involved in nuclear factor κB (NF-κB) activation [10]. Previous studies implicate *UEV1* as a potential proto-oncogene. *UEV1* was initially identified as a transactivator of the *c-fos* promoter [1]. It is downregulated when HT29-M6 colon cancer cells undergo chemical-induced differentiation, and upregulated when Simian virus 40-transformed human embryonic kidney cells become immortal [2]. Furthermore, *UEV1* is variably upregulated in all tumor cell lines examined [4], and maps to chromosome 20q13.2 [3], a region where DNA amplification is frequently reported in breast cancers [11-14] and other tumors [15], as well as in virus-transformed immortal cells [16].

Ubc13-Uev1A is involved in NF-κB activation [10,17,18] and inhibits stress-induced apoptosis in HepG2 cells [19]. Very recently, it was reported that a small-molecule inhibitor of Ubc13-Uev1A interaction can inhibit proliferation and survival of diffuse large B-cell lymphoma cells [20]. These observations collectively establish a close correlation between *UEV1* expression and tumorigenic potential; however, whether *UEV1* plays a role in promoting tumorigenesis or progression and how this is accomplished remains to be elucidated.

NF-кB is a sequence-specific transcription factor known to be involved in innate immunity, anti-apoptosis and inflammation [21-23], and its uncontrolled activation is associated with several types of cancers including breast cancer [24,25]. It regulates a panel of genes that collectively play pro-survival and anti-apoptotic roles [26,27]. It also controls the expression of genes linked with invasion, angiogenesis, and metastasis of cancer, including the matrix metalloproteinase (*MMP*) family [28,29] and chemokine (C-X-C motif) ligand (*CXCL*) family genes [30,31]. Activation of NF-κB is a tightly regulated event. In normal cells, NF-κB becomes activated only after the appropriate stimulation, and then it upregulates the transcription of its target genes [24]. NF-κB is activated by many divergent stimuli, including proinflammatory cytokines such as TNF-α, IL-1b, epidermal growth factor (EGF), T- and B-cell mitogens and bacterial lipopolysaccharides (LPS) [32]. Previous studies reported that the Uev1A-Ubc13 heterodimer is involved in TNF receptor-associated factor 6 (TRAF6) [17,33] and TRAF2-mediated [34] activation of NF-κB, in which Ubc13-Uev1A probably serves as a unique Ubc/E2 along with TRAF proteins to polyubiquitinate NF-κB essential modulator/inhibitor of κB proteinkinase (NEMO/IKKγ) [18,35] and/or Rieske iron-sulfur polypeptide 1(RIP1) [36] to activate IκB kinase (IKK). Activated IKK leads to the phosphorylation and degradation of IκBα, resulting in the release of NF-κB RelA (p65) subunits to translocate into the nucleus [37].

In this study we demonstrate that in MDA-MB-231 breast cancer cells, the *UEV1A* transcript level is moderately elevated compared to normal breast cells. Overexpression of *UEV1A* alone in MDA-MB-231 cells is sufficient to activate NF-кB, which in turn upregulates the *MMP1* expression to enhance breast cancer cell metastasis. More importantly, experimental depletion of Uev1 in MDA-MB-231 cells reduces *MMP1* expression and reduces their ability to grow tumors and metastasize in a xenograft mouse model. These observations provide the experimental and theoretical cornerstone for therapeutic targeting of Uev1A in the treatment of metastatic breast cancers.

## Methods

### Cell culture

Human breast cancer cell lines MDA-MB-231, MCF7, MDA-MB-468, MDA-MB-361, MDA-MB-453, MDA-MB-436 and SK-BRIII were obtained from the American Type Culture Collection (ATCC, Manassan, VA, USA). The cells were cultured in Dulbecco’s minimum essential medium (DMEM) (Invitrogen, Burlington, ON, Canada) supplemented with 10% fetal bovine serum, 100 units/ml penicillin, and 100 μg/ml streptomycin (Invitrogen) in a 5% CO_2_ atmosphere at 37°C. The MCF10A immortalized human mammary epithelial cells were obtained from ATCC and cultured in DMEM/F12 medium supplemented with 10% horse serum, 100 units/ml penicillin, 100 μg/ml streptomycin (Invitrogen), 10 μg/ml insulin (Sigma, St. Louis, MO, USA), 100 ng/ml choleratoxin (Sigma), 0.5 mg/ml hydrocortisone (Sigma), and 20 ng/ml EGF (Peprotech). MDA-MB-231-TR stable cell lines were created by transfecting MDA-MB-231 cell lines with pLenti6-TR-lentivirus (Invitrogen) and selecting with 10 μg/ml blasticidin (Invitrogen).

### Plasmids and pLentivirus vector preparation

Human *MMS2*, *UEV1A*, and *UEV1C* open reading frames (ORFs) were PCR-amplified as *Kpn*I-*Xho*I fragments and cloned into the pcDNA4.0/TO/HA(+) plasmid vector. The 1.9-kb human *MMP1* promoter sequence [GenBank: AJ002550.1] was PCR-amplified as a *Kpn*I-*Hin*dIII fragment and then cloned into the same sites of pGL4.2 (Invitrogen). The NF-кB target site was subsequently mutated by site-directed mutagenesis using a quick-exchange method (Stratagene, La Jolla, CA, USA). The sense primer for creating the NF-кB binding site mutation is 5′-AAAGG CAGAA GGGAA CCTCA *AGAGG TTTT*G AAGAG CCACC GTAAA GTGAG-3′ (mutated sequence italicized). The mutated Ubc13-binding site (F38E) in Uev1A was designed based on a previous study with Mms2-F13E [9]. The modified sequence for *UEV1* small hairpin RNA (shRNA) delivered by lentiviral particles was from Santa Cruz Biotechnology, Inc (Dallas, Texas, USA). The lentiviral particle infection of breast cancer cells was performed following instructions of the supplier. The *MMP1* and *MMP9* small interfering RNAs (siRNAs) were purchased from Genepharma Co Ltd (Shanghai, China). The sequence for *MMP1* siRNA is 5′- GCGUGUGACAGUAAGCUAATT-3′ and that for *MMP9* siRNA is 5′-CGCUCAUGUACCCUAUGUATT-3′.

### RNA preparation and real-time RT-PCR (qRT-PCR)

Total RNA was prepared from cultured breast cancer cells by using TRIzol reagent (Invitrogen). First-strand cDNA was synthesized from total RNA with SuperScript (Invitrogen) according to manufacturer’s instructions. The human breast cancer cDNAs TissueScan™ cancer qPCR Arrays (#BCRT102) were purchased from Origene (Beijing, China). The clinical information is shown on the website [38] and Additional file 1. qRT-PCR analysis was performed on the iQ5 cycler (Bio-Rad, Hercules, CA, USA). The specific primer sets were as follows: *GADPH*, 5′- GAAGGTGAAGGTCGGAGTC-3′ and 5′- GAAGATGGTGATGGGATTTC-3′; *UEV1,* 5′- TCTCCACAGCAATCCTATGAGGTTGA-3′ and 5′- CCAACAGTCGGAAATTGCGAGGG-3′; *UEV1A*, 5′- GAGAGGTTCAAGCGTCTTACCTGAA-3′ and 5′-ACTGTGCCATCTCCTACTCCTTTCT -3′; *UEV1C*, 5′-GCAGCCACCACGGGCTCG-3′ and 5′- CAATTATCATCCCTGTCCATCTTGT-3′; *MMS2*, 5′- CGCTTGTTGGAAGAACTTGA-3′ and 5′- CGGAGGAGCTTCTGGGTAT-3′; *MMP1* 5′- AAATGCAGGAATTCTTTGGG-3′ and 5′-ATGGTCCACATCTGCTCTTG-3′; *MMP9* 5′-CATCGTCATCCAGTTTGGTG-3′ and 5′- TCGAAGATGAAGGGGAAGTG-3′. The relative expression levels were calculated using the comparative cycle threshold (CT) method (2^-ΔCT^) on the Bio-Rad iQ5 (Bio-Rad).

### Luciferase reporter assay

Cells were seeded in 24-well plates at a density of 1 × 10^5^. After 24 hr, the cells were transfected using X-tremeGENE HP DNA Transfection Reagent (Roche, Indianapolis, IN, USA). Briefly, luciferase reporter gene constructs (400 ng), pcDNA-Uevs plasmids (400 ng) and the pRL-SV40 Renilla luciferase construct (5 ng) (for normalization) were co-transfected into the wells. Cell extracts were prepared 48 hr after transfection and the luciferase activity was measured using the Dual-Luciferase reporter assay system (Promega, Madison, WI, USA).

### Western blot analysis

Cells were grown to log phase and lysed in Dulbecco’s PBS (150 mM NaCl, 10 mM Na_2_HPO_4_ and 10 mM NaH_2_PO_4_, pH 7.4) with 1% SDS and the protease inhibitor cocktail for mammalian cells (Sigma-Aldrich). Total protein concentration was determined by the Bradford method using a commercial reagent from Bio-Rad. Cell extracts or purified proteins were electrophoresed in 10% or 15% SDS-polyacrylamide gel electrophoresis (PAGE) gels, transferred to polyvinyl difluoride (PVDF) membrane, and incubated with specific primary antibodies. Monoclonal antibodies (mAbs) LN2B (anti-Uev1) and 4E11 (anti-Ubc13) were from the laboratory stock [10,39]. Primary antibodies against HA (sc-7392), MMP1 (sc-30069), NF-κB (sc-372), Lamin B (sc-166729), β-tublin (sc-6216), and secondary goat anti-mouse antibody IgG-horseradish peroxidase (HRP) (sc-2005) and goat anti-rabbit IgG-HRP (sc-2004) antibody were from Santa Cruz. The P-IκBα antibody (#2859S) was from Cell Signaling Technology (Whitby, ON, Canada), while an anti-MMP9 antibody (ab38898) was from Abcam (Toronto, ON, Canada) and β-actin antibody (BM0627) was from Boster (Wunah, China).

### Immunoprecipitation

We immunoprecipitated 1 mg of protein samples in a total volume of 1 ml with 2 μg of antibody and 20 μl of Protein-A beads (for rabbit polyclonal antibodies) or Protein-G beads (for mouse monoclonal antibodies). The samples were rotated at 4°C overnight. The beads were washed 4 times with 1 ml of cold NP40 lysis buffer containing protease inhibitors. The beads were then boiled for 10 minutes in the presence of 25 μl 2 × sample buffer and the released proteins fractionated by SDS-PAGE in 12% or 15% gels. Proteins were detected by immunoblotting as described above.

### Cell invasion and migration assays

*In vitro* invasion assays were conducted using Transwells (Costar, Cambridge, MA, USA) with 8-μm-pore-size polycarbonate membrane filters in 24-well culture plates. The upper surface of the filter was coated with Matrigel (Becton Dickinson, Bedford, MA, USA) in a volume of 12.5 μl per filter. The Matrigel was dried and reconstituted at 37°C into a solid gel on the filter surface. The lower surface of the filter was coated in fibronectin (20 μg/ml), vitronectin (10 μg/ml), collagen IV (50 μg/ml), or 10% (BSA)-DMEM as chemoattractants. After starving in BSA-free DMEM overnight, 2 × 10^4^ cells were seeded in the upper chamber. The cells were allowed to invade for 48 hr. Cells that invaded the lower surface of the filter were counted in five random fields under a light-microscope at high magnification. Experiments were conducted at least in triplicate. The *in vitro* cell migration ability was detected by wound healing assay or transwell assay without Matrigel coating. The wound healing assay was performed by seeding 2 × 10^5^ cells onto 96-well plates. Confluent monolayers were wounded using a pipette tip. After 48 hr the cell migration distance was measured under a light-microscope at high magnification in at least three random fields. The transwell assay without Matrigel was conducted using Transwells with 8-μm-pore-size polycarbonate membrane filters in 24-well culture plates. The lower surface of the filter was coated with 10% BSA-DMEM as chemoattractants. After starving in a BSA-free DMEM medium overnight, 5 × 10^4^ cells were seeded in the upper chamber. Cells were allowed to invade for 6 to 36 hr and those migrated to the lower surface of the filter were counted in five random fields under a light-microscope at high magnification. Experiments were conducted at least in triplicate.

### Metastasis assay in a xenograft mouse model

The experimental mouse work followed the animal care protocol CNUAREB-2012002 approved by the Capital Normal University Animal Research Ethics Board and was conducted at the Peking University Health Science Center, China. For the tumorigenesis assays, 5 × 10^6^ breast cancer cells were injected subcutaneously into the lateral flanks of 4- to 5-week-old BALB/c female nude mice. The palpable tumor diameters were measured once per week. Tumor length (L) and width (W) were measured with a caliper, and the volume (V) was calculated by the following equation:

$$V=\left({L\times W}^{2}\right)/2.$$

The mice were sacrificed 6 weeks after cell injection. For experimental metastasis assays by intravenous (i.v.) injection, 2 × 10^5^ breast cancer cells were injected into the tail veins of the 4- to 5-week-old female BALB/c nude mice. Endpoint assays were conducted 5 weeks after injection. Metastatic lung nodules 0.5 mm in diameter were counted. Analysis of variance (ANOVA) was used for statistical analyses. To ensure representative sampling of lung tumor nodules, four sections were made per lung at various depths along the coronal plane of the lung. The nodules per lung (four sections) were counted under a light-microscope.

### Histopathology

Formalin-fixed lungs were paraffin-embedded, and tissue sections derived from tumor nodules or other tissues were stained with H&E to evaluate the morphology and invasiveness of breast cancer cells. Metastatic tumor nodules were counted throughout the entire lung section at all three depths under a light-microscope. Anti-Uev1A (LN1), anti-MMP1 (sc-30069) and NF-κB p65 (sc-372) primary antibodies from Santa Cruz were used for immunohistochemistry (IHC). TissueFocus™ breast cancer tissue microarrays (CT565863) for IHC were obtained from Origene (Beijing, China). Microscopic images were captured by a SPOT digital camera mounted in a light-microscope.

### Preparation of nuclear fraction

HeLa cells were treated with 40 ng/ml TNF-α for 2 hr. Cells were washed, scraped with PBS, and centrifuged at 3,000 rpm at 4°C. The pellet was suspended in 10 mM Tris (pH 8.0) with 1.5 mM MgCl_2_, 1 mM dithiothreitol, and 0.1% NP-40, and incubated on ice for 15 minutes. Nuclei were separated from cytosol by centrifugation at 12,000 rpm at 4°C for 15 minutes. The cytosolic supernatants were removed and the precipitated pellets were suspended in 10 mM Tris (pH 8.0) containing 100 mM NaCl and stored on ice for 30 minutes. After agitation for 30 minutes at 4°C, the lysate was centrifuged at 12,000 rpm for 15 minutes at 4°C, and the supernatant was collected.

### Electrophoretic mobility shift assay (EMSA)

The secquence of biotin-labelled sense NF-κB probe for EMSA is 5′-GAACCTCAGAGAACCCCGAAGAGCC-3′. The cold probe is the same NF-κB sequence without biotin label. The sequence of biotin-labelled mutated sense NF-κB probe is 5′-GAA CCTCA *AGAGGTTTT*G AAGAGCC-3′ (mutated sequence underlined): 1 ng of the probe was incubated together with 10 to 20 μg of cell extracts or 5 to 10 μg of nuclear extracts for 30 minutes at 25°C in a final volume of 20 μl. The binding reaction was subsequently separated on a 5.5-7% poly-acrylamide gel in 1x Tris-Borate-EDTA (TBE) buffer (90 mM Tris, 90 mM boric acid).

### Statistical analysis

The statistical significance of differential findings between the experimental and control groups was determined by Student’s *t*-test as implemented by Microsoft Excel 2010 (*P <0.05, **P <0.01 and ***P <0.001).

## Results

### Alternative *UEV1* transcript levels in breast cancer cell lines and samples

**Figure 1:**
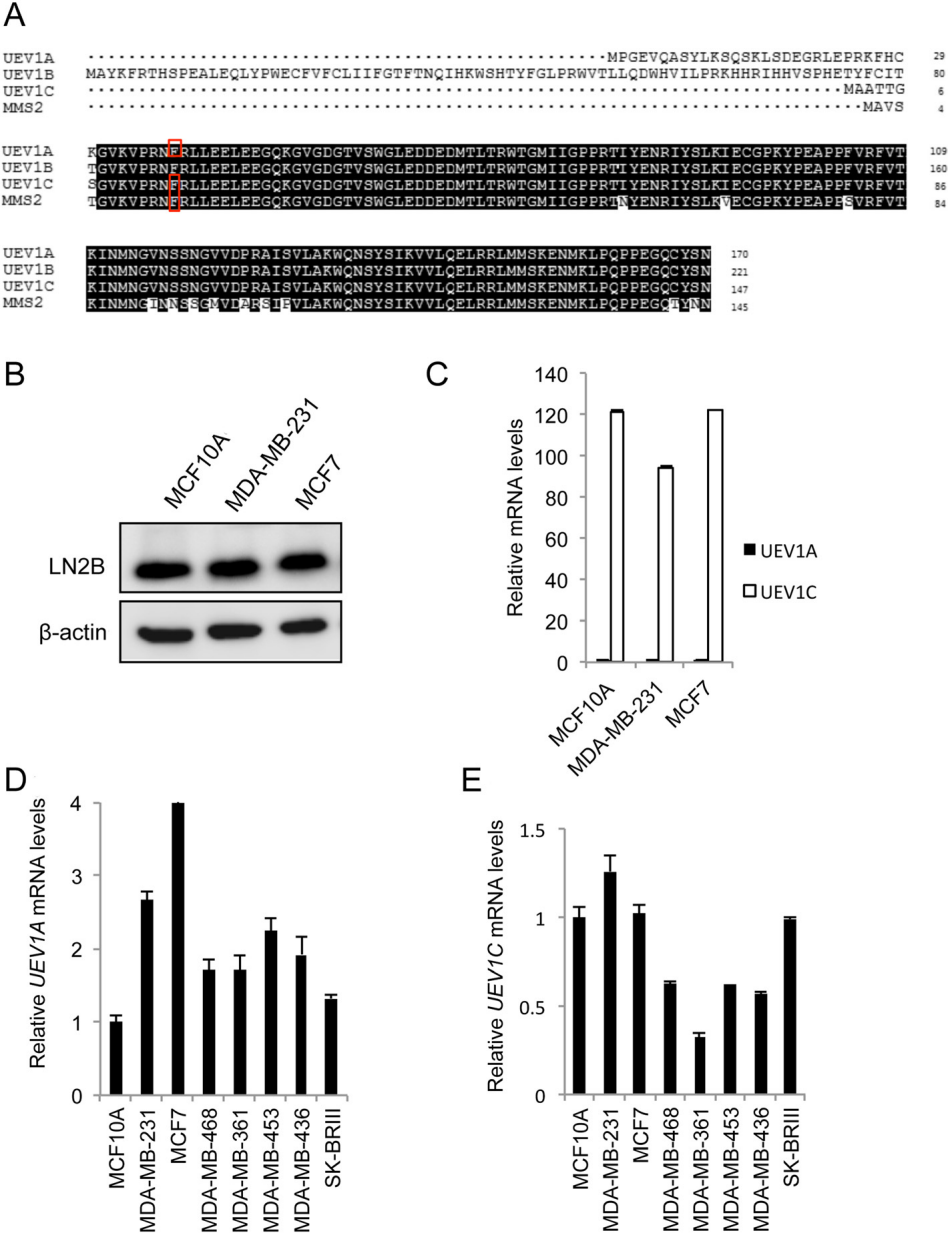
***Ubiquitin conjugating enzyme variant *(*UEV*) *1A *is overexpressed in breast cancer cell lines and tumor samples. (A)** Amino acid sequence alignment of ubiquitin conjugating enzyme variant (Uev)1A, Uev1B, Uev1C and Mms2. Residues critical for the interaction with Ubc13 [9] are indicated with a red box. **(B)** Uev1 variant protein levels in human breast cancer cell lines. Whole cell extracts were analyzed by western blotting with the anti-Uev1 monoclonal antibody LN2B [39]. **(C)** Relative transcript levels of *UEV1* variants in human breast cancer cell lines as determined by qRT-PCR. **(D)** Relative *UEV1A* transcript levels in human breast cancer cell lines as determined by qRT-PCR. **(E)** Relative *UEV1C* transcript levels in human breast cancer cell lines as determined by qRT-PCR.

Two major human *UEV1* transcripts (*UEV1A* and *UEV1B*) were previously reported [1]. It was determined that only Uev1A, but not Uev1B, is able to physically interact with Ubc13 and promote K63-linked polyubiquitination [10]. It turns out that Uev1B is excluded from the nucleus and involved in endosomal trafficking [40]. Interestingly, database analyses also indicate another splicing variant that would encode a 147-amino acid Uev1 core domain, which we name Uev1C (Figure 1A), the cellular function of which is currently unknown.

Western blot analysis of endogenous Uev1s using a Uev1-specific monoclonal antibody LN2B [39] could only detect Uev1C in several breast cancer cell lines including MDA-MB-231 and MCF7, and MCF10A, an immortalized normal mammary epithelial cell line (Figure 1B). qRT-PCT analysis revealed that the relative transcript level of *UEV1C* is approximately 100-fold higher than that of *UEV1A i*n the above cell lines (Figure 1C), consistent with observations in other cell lines including HeLa and U2OS (data not shown).

We next examined relative transcript levels of *UEV1A* and *UEV1C* in breast cancer lines using MCF10A as a reference. Interestingly, the *UEV1A* transcript level is elevated in all breast cancer cell lines examined (Figure 1D), with no significant upregulation of *UEV1C* (Figure 1E) or *MMS2* (Additional file 2: Figure S1A) in these lines. It has been previously reported that the *UEV1A* level may be elevated when normal cells undergo immortalization [2]. To further assess relative *UEV1A* expression in normal versus breast tumor tissues, we measured the *UEV1A* transcript level in five normal human breast samples and 43 breast cancer samples from TissueScan microarrays. Compared with the five normal human breast samples, 33/43 or 77% of breast cancer samples display *UEV1A* expression above the highest *UEV1A* level in normal samples, or at least 1.7-fold higher than the average level among the five normal samples (Additional file 2: Figure S1B), suggesting that Uev1A may play a role in promoting breast tumorigenesis.

### Overexpression of *UEV1A* promotes breast cancer cell invasion *in vitro* and metastasis in a xenograft model

**Figure 2:**
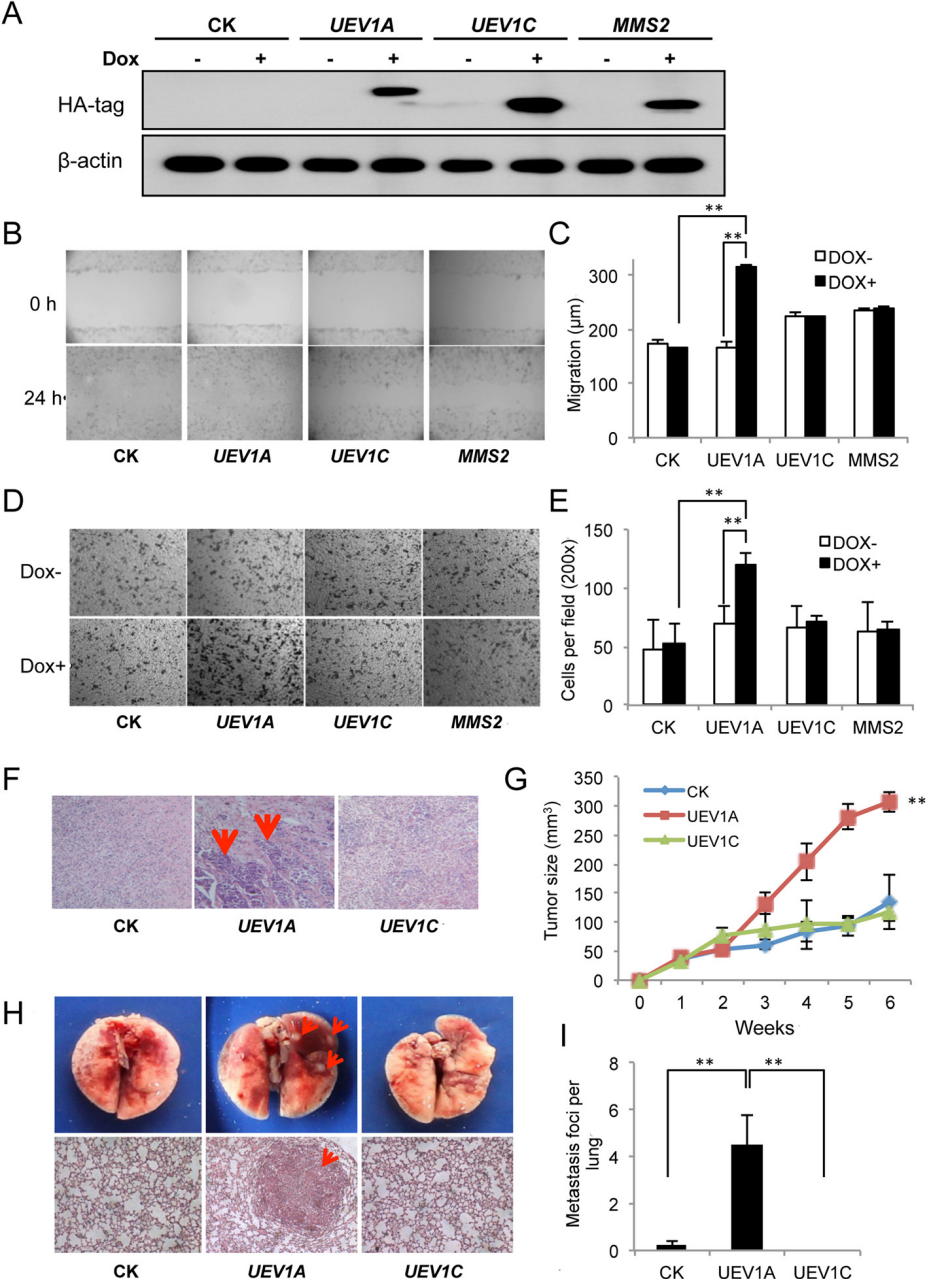
***UEV1A *overexpression promotes breast cancer cell invasion *in vitro *and metastasis in a xenograft model. (A)** pcDNA4.0/TO/HA(+) vector (CK) expressing *UEV1A*, *UEV1C* or *MMS2* was stably transfected into MDA-MB-231-TR cells, with or without doxycycline (Dox) treatment. The level of ectopic gene expression was monitored by western blot against an anti-HA antibody. **(B)** Representative images of wound-healing assays with Dox treatment. **(C)** Statistical analysis of cell migration of wound-healing assay with and without Dox treatment. The migration distance of cells was measured in five different wells in each group under a light-microscope. **(D)** Representative images of cell invasion assay with Matrigel-coated transwells. **(E)** Statistical analysis of the cell invasion assay data. Cells that invaded the lower surface of the filter were counted in five random fields under a light-microscope at 200× magnification. **(F-I)***In vivo* tumorigenesis and metastasis assays using a xenograft mouse model. **(F)** Lymph node sections after sacrifice were stained with H&E. The lymph node metastasis sites are shown by red arrows. **(G)** Quantitative analysis of tumor growth. Tumor growth was measured every week after injection (Day 0) and expressed as mean ± SD (n = 10). **(H)** The *in vivo* metastasis assay in xenograft mice. Upper panel, the lung metastasis nodules formed are shown by red arrows. Lower panel, the lung sections were stained with H&E and the lung metastasis under a light-microscope at 100× magnification is indicated by a red arrow. **(I)** Quantitative analysis of the *in vivo* lung metastasis as measured by the number of metastasis foci per lung for all four sections (n = 10 mice for each treatment).

To ask whether an elevated *UEV1A* level is indeed sufficient to promote breast cancer, *UEV1A*, *UEV1C* or *MMS2* genes were cloned into a pcDNA4.0/TO/HA(+) vector and then transfected into MDA-MB-231-TR cells to construct stable cell lines, and the level of ecotopic gene expression after 10 μg/ml doxycycline (Dox) treatment was monitored by qRT-PCR (Additional file 2: Figure S2A-C) and western blot against the HA tag (Figure 2A), Uev1 (LN2B, Additional file 2: Figure S2D) or Uev1 plus Mms2 (LN3, Additional file 2: Figure S2E).

The cell growth and cell cycle progression of stable MDA-MB-231 transfectants were first measured, with no obvious alterations among each group (Additional file 2: Figure S3). The effects of ecotopic genes on breast cancer cell migration and invasion were then measured. The wound-healing experimental data show that the overexpression of *UEV1A* doubles the mobility compared with the same cells without ecotopic *UEV1A* expression or vector-transfected MDA-MB-231-TR cells with Dox treatment, while overexpression of *UEV1C* or *MMS2* does not affect cell mobility compared with uninduced cells (Figure 2B, C and Additional file 2: Figure S4). In a transwell assay, the invasiveness of *UEV1A* transfectants after induction was approximately 2.3-fold higher than the control, *UEV1C* or *MMS2* transfectants, whereas there was no significant difference among control, *UEV1C* and *MMS2* transfectants regardless of Dox treatment (Figure 2D and E). These results suggest that *UEV1A* regulates breast cancer cell migration and invasion *in vitro*.

As an increased ability of cancer cells to migrate and invade *in vitro* is a faithful indicator of cell metastasis, to further confirm the correlation between *UEV1A* expression and breast cancer metastasis, we assessed the effects of *UEV1A* on metastasis using an *in vivo* xenograft mouse model. Stably-transfected MDA-MB-231-TR cells were injected into the lateral flanks of 4- to 5-week-old BALB/c female nude mice and Dox (625 mg/kg) was added in feed as soon as the cells were injected. Tumor growth and metastasis were then monitored. All mice (10/10) injected with the *UEV1A*-expressing cells had massively enlarged lymph nodes containing invasive breast cancer cells (Figure 2F). In contrast, there were no tumor metastasis foci in lymph nodes of mice injected with vector control or *UEV1C*-expressing cells, although some lymph nodes were enlarged (data not shown). Furthermore, overexpression of *UEV1A* but not *UEV1C* accelerated tumor growth compared to vector-transfected cells (Figure 2G). In the tail-vein injection groups, compared with *UEV1C* or vector control, overexpression of *UEV1A* significantly promoted lung metastasis colony fomation (Figure 2H, and I). These observations collectively demonstrate that elevated expression of *UEV1A* alone is sufficient to promote tumor growth and metastasis.

### Depletion of Uev1 prevents breast cancer cell invasion *in vitro* and metastasis in nude mice

**Figure 3:**
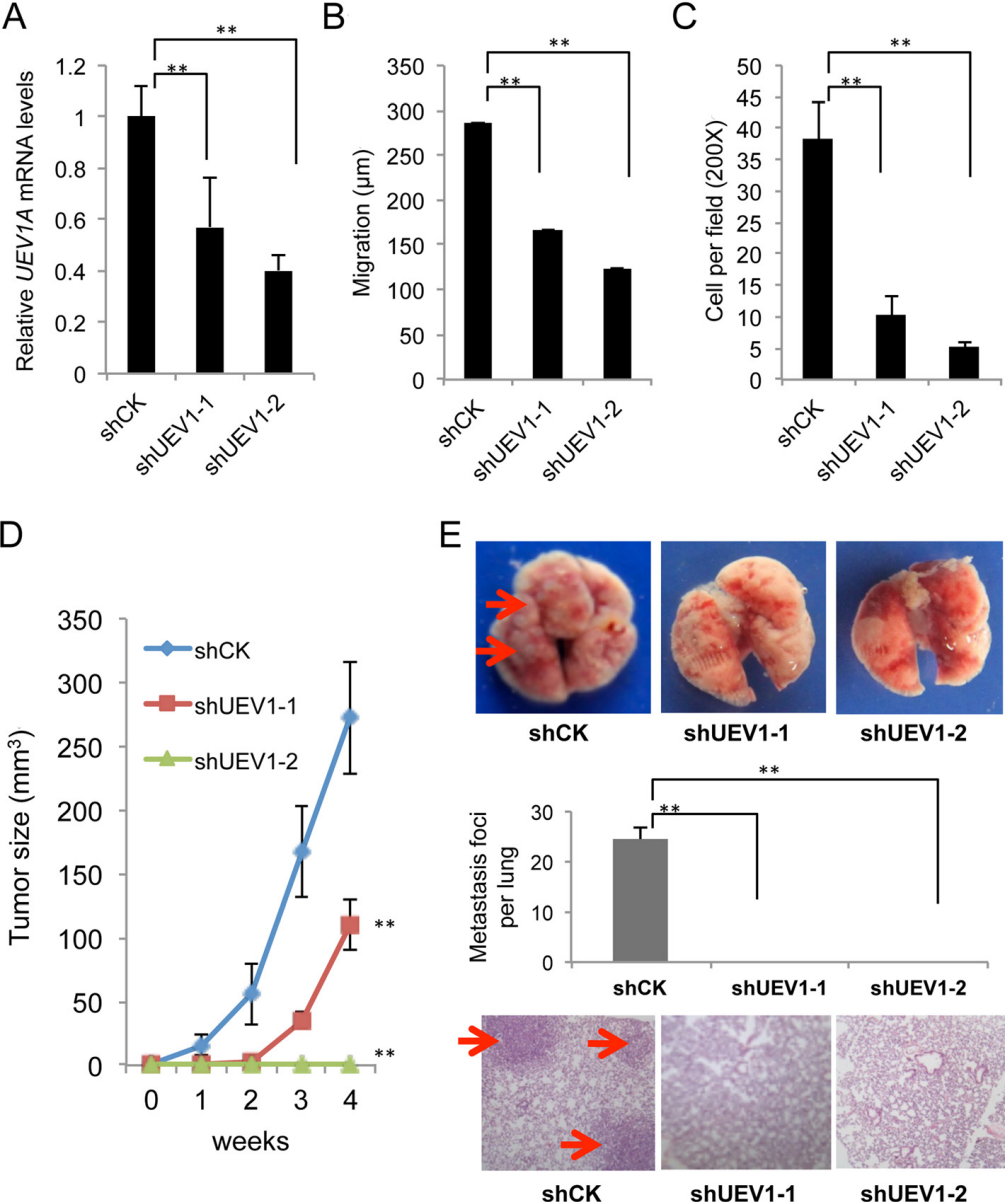
**Depletion of ubiquitin conjugating enzyme variant (Uev)1 reduces cell invasion *in vitro *and metastasis in a xenograft mouse model. (A)** MDA-MB-231 cells were transfected with shRNA lentiviral particles against *UEV1* (sh*UEV1*) or non-specific target (shCK). shUEV1-1 and shUEV1-2 represent two independent stable shUEV1 lines. *UEV1A* transcript levels in shCK and shUEV1 lines were determined by qRT-PCR. **(B)** Quantitative analysis of cell migration by wound-healing assay. The migration distance of cells was measured in five different wells in each treatment group under a light-microscope. **(C)** Quantitative analysis of cell invasion in Matrigel-coated transwells. Cells invading the lower surface of the filter were counted in five random fields under a light-microscope at 200× magnification. **(D, E)** The *in vivo* tumorigenesis assay using a xenograft mouse model. 1 × 10^6^ MDA-MB-231 cells depleted with shUEV1 or shCK were injected into the lateral flanks of 4- to 5-week-old BALB/c female nude mice. **(D)** Tumor growth was measured every week after injection (Day 0) and expressed as mean ± SD (n = 10). **(E)** Upper panel, lungs from mice injected via tail veins with MDA-MB-231 cells treated with shUEV1s or control. Red arrows point to lung metastasis foci. Middle panel, quantitative analysis of the number of metastasis foci per lung. The nodules per lung for all four sections were counted under a light-microscope (×100) (n = 10 mice for each treatment). Lower panel, sample lung sections stained with H&E. Red arrows point to lung metastasis foci. All samples were taken after sacrifice.

Compared with MCF-10A cells, there was a 2.7-fold increase in the *UEV1A* expression in MDA-MB-231 cells (Figure 1D). To ask whether this moderate overexpression of *UEV1A* contributes to breast cancer metastasis, the endogenous *UEV1A* expression in MDA-MB-231 cells was suppressed using an shRNA (shUEV1) delivered by lentiviral particles. It was found that two independent shUEV1 constructs, shUEV1-1 and shUEV1-2, reduced *UEV1A* expression to 40% and 55% of control shRNA-treated cells, respectively (Figure 3A). As expected, the cellular *UEV1C* mRNA and protein levels were also reduced but *MMS2* remained unaffected (Additional file 2: Figure S5A-C). Moreover, partial depletion of Uev1 reduced cell migration (Figure 3B and Additional file 2: Figure S5D) and invasion (Figure 3C and Additional file 2: Figure S5E). The above findings were further extended by using a xenograft lung metastasis model, in which depletion of Uev1 limited tumor growth to the extent that no tumor was found in nude mice injected with MDA-MB-231 cells in which the Uev1 level was reduced by shUEV1-2 (Figure 3D and Additional file 2: Figure S5F). Furthermore, depletion of Uev1 in MDA-MB-231 cells significantly reduced the number of lung nodules formed in mice and completely abolished metastasis in lung, even with moderate depletion of Uev1 (Figure 3E, shUEV1-1, middle panel). These results clearly indicate that the elevated *UEV1A* expression in MDA-MB-231 cells plays a critical role in breast tumorigenesis and metastasis.

### Overexpression of *UEV1A* activates NF-κB in MDA-MB-231 cells in a Ubc13-dependent manner

**Figure 4:**
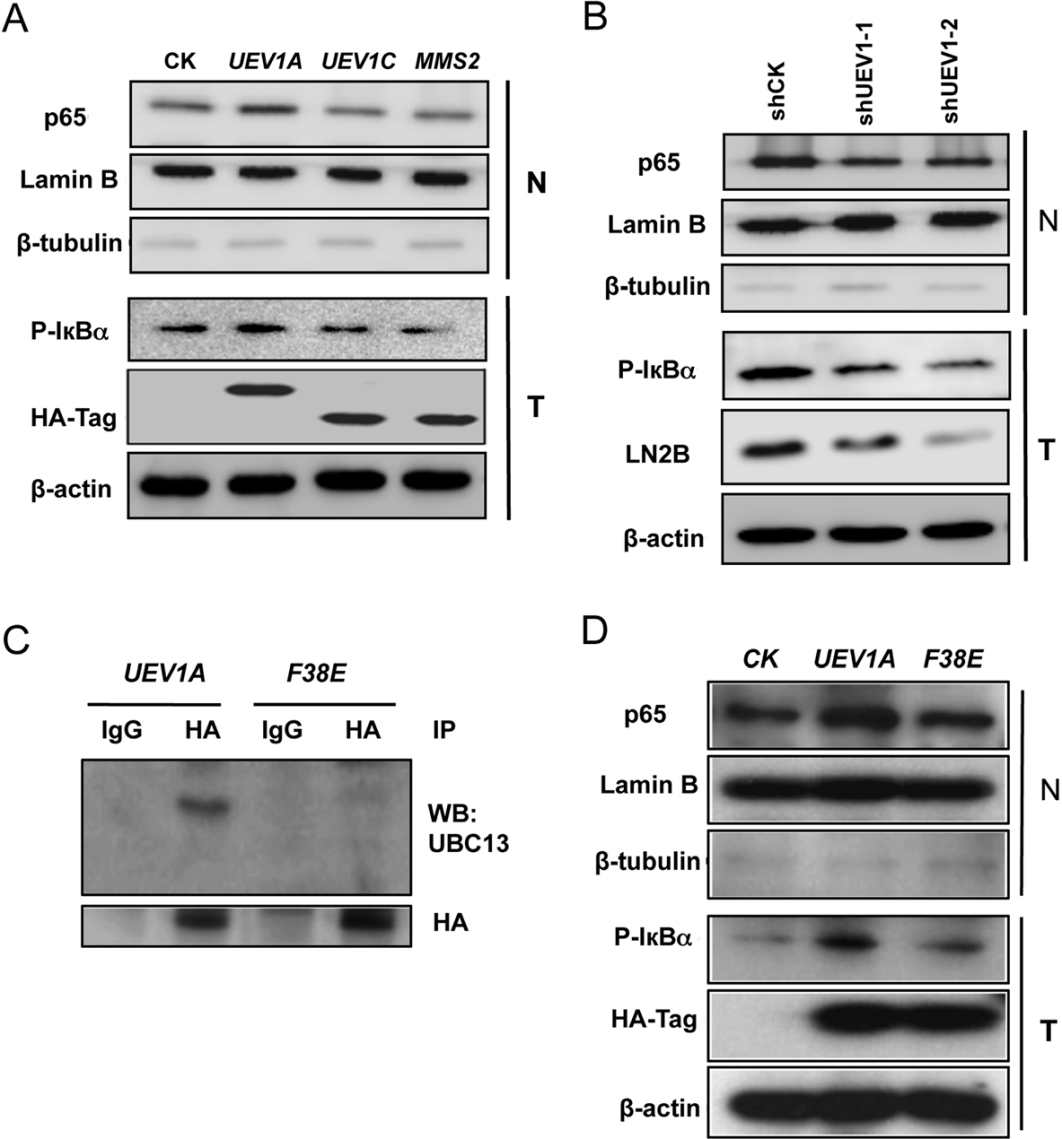
**Ubiquitin conjugating enzyme variant (Uev)1A activates NF-κB in MDA-MB-231 cells in a Ubc13-dependent manner. (A)** NF-κB activation in *UEV*-overexpressing cells. Nuclear or whole-cell extracts were prepared, equal amounts of protein were separated by SDS–PAGE gel, followed by western blotting analysis using an anti-p65 antibody to measure NF-кB nuclear enrichment and an anti-P(S32)-inhibitor of NF-κBα (IκBα) antibody to assess the degree of IκBα phosphorylation as an indication of its degradation and release of NF-κB into the nucleus. **(B)** NF-κB activation in Uev1-depleted cells. Experimental conditions are as described in Figure 4A. **(C)** The F38 residue of Uev1A was required for its interaction with Ubc13. MDA-MB-231 cell extracts expressing *UEV1A* or *UEV1A-F38E* were immunoprecipitated with an anti-HA monoclonal antibody, followed by western blotting with an anti-Ubc13 monoclonal antibody and an anti-HA monoclonal antibody. **(D)** Uev1A-F38E failed to activate NF-κB. Experimental conditions were as described in Figure 4A.

To understand the mechanism by which Uev1A promotes metastasis in breast cancer cells, we took into account that Uev1A has been reported to activate NF-κB in HepG2 [19], and that NF-κB regulates the expression of a large number of genes critical for tumorigenesis, inflammation and metastasis [41]. As a hallmark of NF-κB activation is its translocation from the cytoplasm to the nucleus, we transfected MDA-MB-231-TR cells with a variety of constructs, induced the target gene expression by adding Dox, fractionated cells and then measured the subcellular distribution of the p65 subunit of NF-κB. As seen in Figure 4A, only overexpression of *UEV1A*, but not *UEV1C* or *MMS2*, was able to increase the phosphorylation of the NF-κB inhibitor IκBα (presumably in the cytoplasm) and enrich p65 in the nucleus. Consistently, depletion of Uev1 by shRNA reduced IκBα phosphorylation and p65 nuclear translocation (Figure 4B).

It has been previously reported that Uev1A is a cofactor of Ubc13 in the NF-κB signaling pathway [10,17]. To ask whether the above Uev1A function is indeed dependent on Ubc13, we created a Uev1A-F38E mutation as the corresponding Mms2-F12E mutation (Figure 1A) absolutely abolishes its interaction with Ubc13 and its ability to promote Ubc13-mediated K63 polyubiquitination [9]. Indeed we confirmed that the Uev1A-F38E substitution does not affect its expression (Additional file 2: Figure S2F) but abolishes its interaction with Ubc13 *in vivo* (Figure 4C). As expected, overexpression of *UEV1A-F38E* failed to activate NF-κB, as judged by a lack of IκBα phosphorylation and p65 nucleaar translocation (Figure 4D).

### *MMP1* and *MMP9* are tightly regulated by *UEV1*

**Figure 5:**
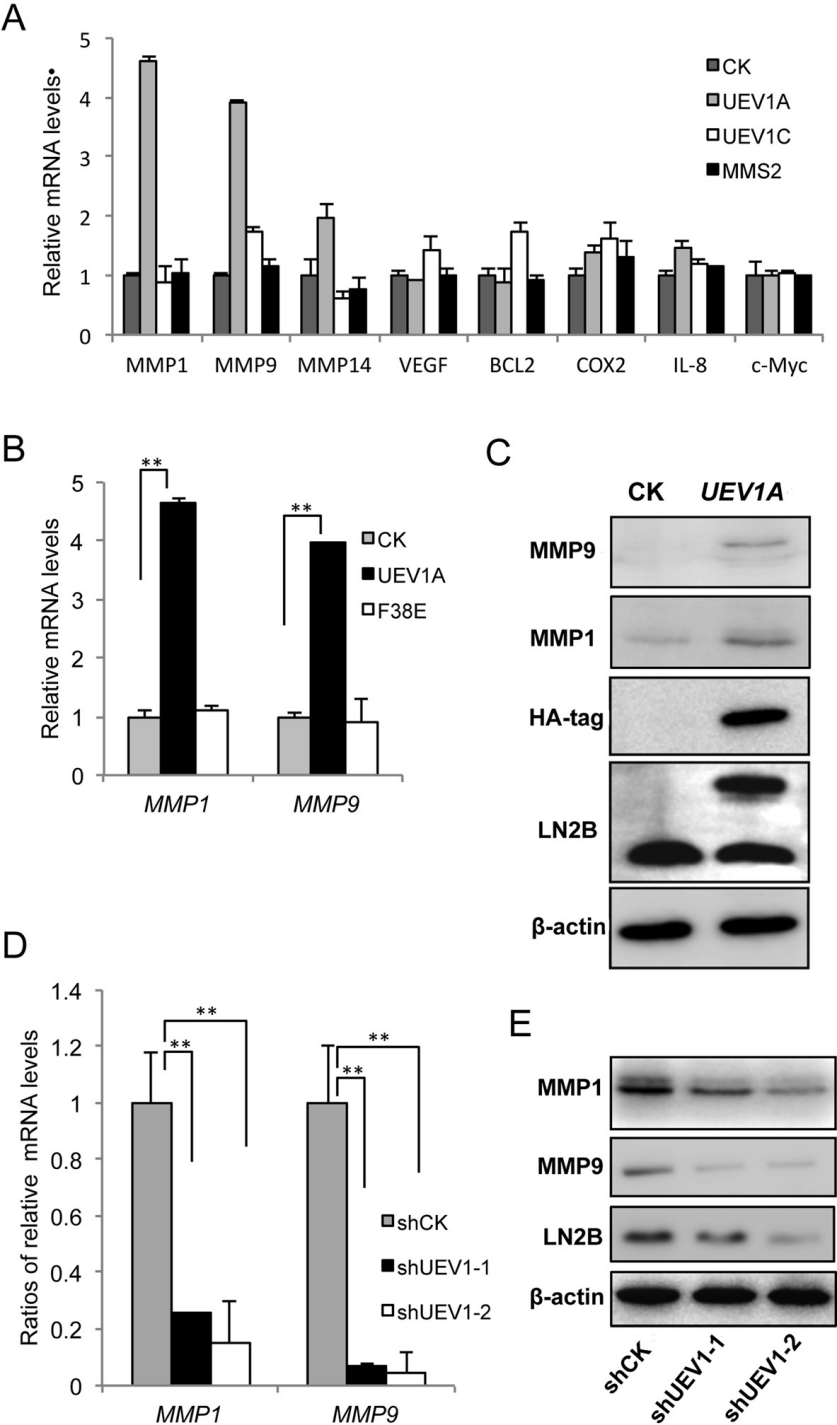
**Ubiquitin conjugating enzyme variant (Uev)1A positively regulates *MMP1 *and *MMP9 *expression. (A)** The transcript levels of selected putative NF-κB target genes in MDA-MB-231-TR cells expressing different *UEV*s as determined by qRT-PCR. **(B)** Overexpression of *UEV1A* but not *UEV1A-F38E* stimulated *MMP1* and *MMP9* expression in MDA-MB-231 cells as determined by qRT-PCR. **(C)** Elevated MMP1 and MMP9 protein levels in *UEV1A*-overexpressed MDA-MB-231 cells as determined by western blot. **(D)** The transcript levels of *MMP1* and *MMP9* in two independent shUEV1-transfected MDA-MB-231 cell lines as determined by qRT-PCR. **(E)** MMP1 and MMP9 protein levels in two independent shUEV1-transfected MDA-MB-231 cell lines as determined by western blot.

As NF-κB is a transcriptional factor that regulates the expression of a large number of genes including those involved in metastasis, we measured the transcription of many established or putative NF-κB target genes thought to be involved in metastasis such as *COX2*[42], *VEGF*[43] and *MMP* family genes, among which *MMP1* and *MMP9* transcripts were elevated by 4.6- and 3.9-fold, respectively, in *UEV1A*-overexpression cells, but not in *UEV1C or MMS2* overexpression cells (Figure 5A,B), with corresponding elevation at proteins levels (Figure 5C). The observed increase is completely dependent on Ubc13, as overexpression of *UEV1A-F38E* failed to induce *MMP1* or *MMP9* (Figure 5B). Similarly, depletion of *UEV1* in MDA-MB-231 cells significantly reduced *MMP1* and *MMP9* transcript (Figure 5D) and protein (Figure 5E) levels, and more efficient depletion of *UEV1* (shUEV1-1 versus shUEV1-2) resulted in stronger repression of *MMP1* and *MMP9* expression (Figure 5D and E), indicating that *MMP1* and *MMP9* are tightly regulated by Uev1A-Ubc13.

### MMP1 is a downstream effector for Uev1A-induced metastasis

**Figure 6:**
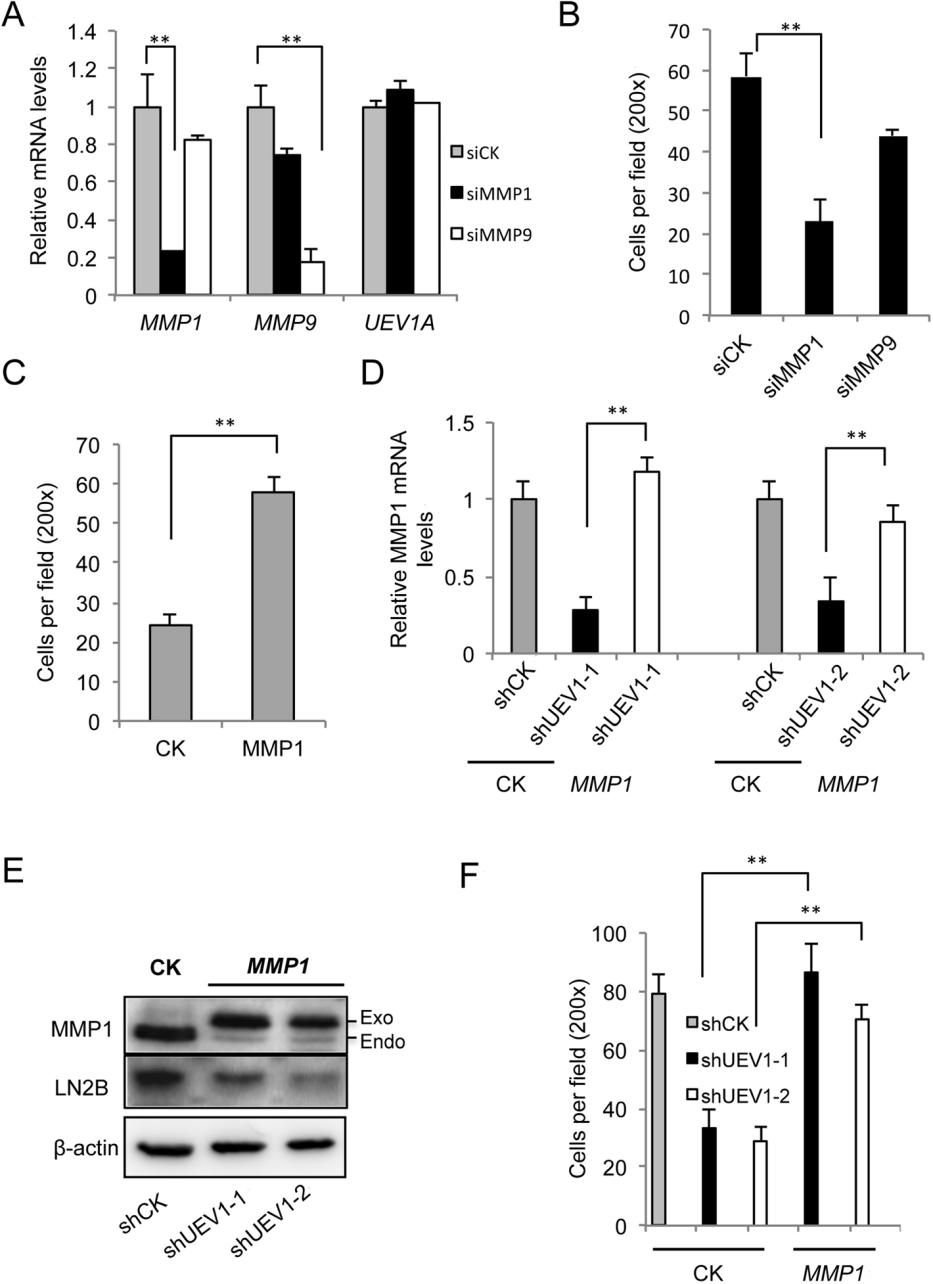
**Matrix metalloproteinase (MMP)1 is a downstream effector for ubiquitin conjugating enzyme variant (Uev)1A-induced metastasis. (A)** The mRNA levels of *MMP1*, *MMP9* and *UEV1A* in *MMP1* or *MMP9* knocked-down MDA-MB-231 cells as determined by qRT-PCR. **(B)** Quantitative analysis of cell invasive ability in Matrigel-coated transwells. MDA-MB-231 cells depleted of MMP1 or MMP9 were subject to the transwell assay and at least five random fields were counted under a light-microscope at 200× magnification. **(C)** Quantitative analysis of cell invasive ability in Matrigel-coated transwells. MDA-MB-231 cells overexpressing *MMP1* were subject to the transwell assay and at least five random fields were counted under a light-microscope at 200× magnification. **(D)** Ectopic expression of *MMP1* restored the *MMP1* transcript level in two independent *UEV1*-depleted cell lines as determined by qRT-PCR. **(E)** Ectopic expression of *MMP1* restored the MMP1 protein level in two independent *UEV1*-depleted cell lines as determined by western blot. **(F)** Quantitative analysis of cell invasive ability in Matrigel-coated transwells. At least five random fields were counted under a light-microscope at 200× magnification.

To ask whether MMP1 and/or MMP9 are critical effectors for Uev1A-induced metastasis, we depleted *MMP1* or *MMP9* by siRNA in MDA-MB-231 cells (Figure 6A). The above treatment did not affect the *UEV1A* expression (Figure 6A), but the depletion of *MMP1* significantly decreased the invasiveness of MDA-MB-231 cells as determined by a transwell assay, while *MMP9* depletion had much less effect (Figure 6B and Additional file 2: Figure S6A). Similarly, *MMP1* depletion in *UEV1A*-overexpressed MDA-MB-231 cells (Additional file 2: Figure S6B) also decreased invasiveness (Additional file 2: Figure S6C and D). To ask whether overexpression of *MMP1* is indeed sufficient to dictate MDA-MB-231 cell invasion, we constructed an *MMP1*-expression plasmid and transfected it to MDA-MB-231 cells, which resulted in a 2.6-fold increase in the *MMP1* transcript level (Additional file 2: Figure S6E) and a similar increase at the protein level (Additional file 2: Figure S6F), as well as a 2.3-fold increase in invasion (Figure 6C and Additional file 2: Figure S6G). We then restored *MMP1* level in *UEV1*-depleted cells to that of control cells (Figure 6D and E), which was sufficient to rescue the invasiveness in both *UEV1*-depleted MDA-MB-231 cell lines (Figure 6F). As *MMP1* is an important cancer cell metastasis factor [44-46], the above findings allow us to conclude that *UEV1A* regulates metastasis through tightly controlling *MMP1* expression.

### UevA-Ubc13 control *MMP1* expression by regulating NF-κB

**Figure 7:**
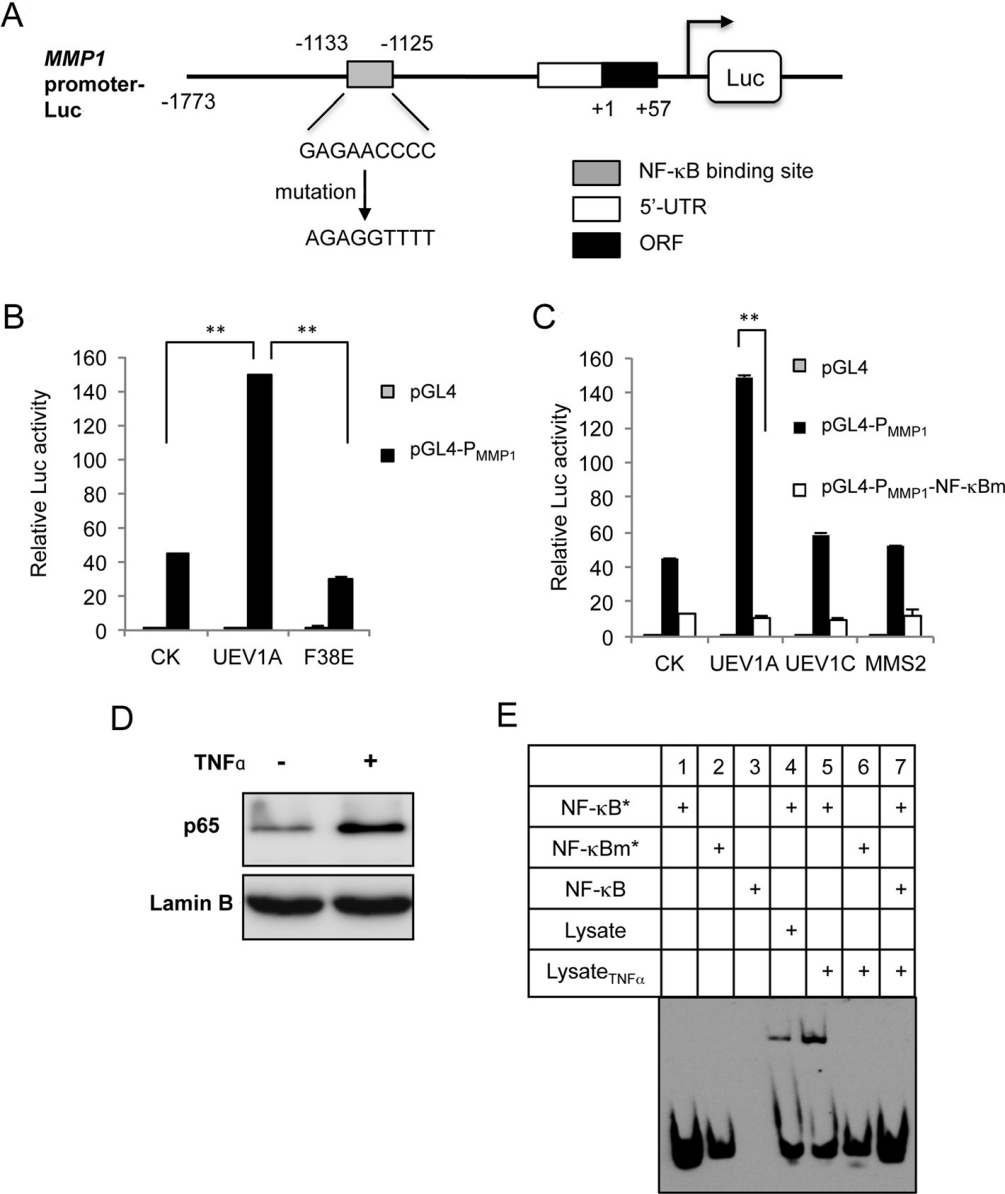
**Ubiquitin conjugating enzyme variant (Uev)1A regulates *Matrix metalloproteinase 1 *(*MMP1*) expression through an NF-κB target sequence in the *MMP1 *promoter. (A)** A schematic illustration of the *MMP1* promoter and the P_*MMP1*_-Luciferase (Luc) reporter construct. A putative NF-κB binding site in the *MMP1* promoter [GenBank: AJ002550.1] is mapped to 1133–1125 nucleotides upstream of its start codon. The mutated sequence in the P_*MMP1*_-NF-κBm construct is also shown. **(B)** The P_*MMP1*_-Luc reporter was co-transfected to MDA-MB-231 cells with constructs that overexpressed wild-type *UEV1A* or *UEV1A-F38E*. The data were normalized to the activity of cells transfected with the empty vector (pGL4.2). **(C)** Only *UEV1A*, but not *UEV1C* or *hMMS2*, was able to activate the *MMP1* promoter, and this ability was dependent on its interaction with Ubc13. The experimental conditions were as described in Figure 7B. **(D)** TNFα activates NF-κB as measured by the nuclear localization of the p65 subunit of NF-κB. HeLa cells were treated with or without TNFα for 30 minutes, nuclear proteins were purified and the relative p65 level in the nuclear fraction was determined by western blot and the nuclear protein Lamin B was used as a loading control. **(E)** Physical interaction between NF-κB and the putative NF-κB target sequence in the *MMP1* promoter as determined by electrophoretic mobility shift assay. Lane 1, biotin-labeled NF-κB target sequence probe (NF-κB*). Lane 2, biotin-labeled mutated NF-κB binding site probe (NF-κBm*). Lane 3, Unlabeled double-strand DNA containing the P_*MMP1*_ NF-κB target sequence (NF-κB). Lanes 4 to 7 contain either wild-type or mutated P_*MMP1*_ NF-κB target sequence as indicated in the upper panel, plus TNFα-treated or untreated cell extract. Lane 7 also contains excessive unlabeled DNA of P_*MMP1*_ NF-κB target sequence.

To ask whether Uev1A regulates *MMP1* through NF-κB, we cloned the 1.8-kb human *MMP1* promoter sequence (Figure 7A) into pGL4.2 and co-transfected it with plasmids expressing *UEV1A*, *UEV1A-F38E* or an empty vector into MDA-MB-231 cells. A luciferase assay showed that *UEV1A* expression activates the *MMP1* promoter and that this activation relies on its interaction with Ubc13, as the Uev1A-F38E substitution completely abolished the activation (Figure 7B). The predicted NF-κB binding site (−1133 to approximately −1125) in the *MMP1* promoter was then mutated (Figure 7A) and the mutated reporter was used to co-transfect with plasmids expressing *UEV1A*, *UEV1C*, *MMS2* or the empty vector into MDA-MB-231 cells. Overexpression of only *UEV1A*, but not *UEV1C* or *MMS2*, activated the wild-type *P*_*MMP1*_*-Luc* reporter and this activation absolutely required the intact NF-κB target site (Figure 7C). To gain direct evidence that the predicted NF-κB binding site indeed interacts with NF-κB, an EMSA was performed using nuclear lysates from TNFα-treated and untreated cells (Figure 7D). A biotin-labeled synthetic *MMP1* promoter probe containing the putative NF-κB-binding sequence was able to interact with the nuclear lysate and this interaction was enhanced when cells were pretreated with TNF-α, which induced nuclear translocation of p65 (Figure 7E, lanes 4 and 5). This interaction was abolished when the NF-κB binding sequence was mutated (lane 6) or out-competed by adding excess unlabeled probe (lane 7), indicating that the interaction is sequence-specific. We conclude from the above observations that NF-κB directly binds to the *MMP1* promoter at the predicted binding site.

## Discussion

It has been reported previously that human cells contain two *UEV* genes, *UEV1* and *MMS2*, which share >90% amino acid sequence-identity in their core domains [4]. Although both Uev1A and Mms2 proteins serve as cofactors for Ubc13-mediated K63-linked polyubiquitination, their biological functions are apparently distinct and only the Uev1A-Ubc13 complex is involved in NF-κB signaling [10]. It has been puzzling us that Uev1A and Mms2 have different molecular weights and migrate differently; however, a monoclonal antibody capable of recognizing both purified Uev1A and Mms2 only detects a single band in western blot analysis, and siRNA depletion of either Mms2 or Uev1 only partially reduced the intensity of this band. A careful examination in this study reveals that in addition to the previously reported two *UEV1* splicing variants *UEV1A* and *UEV1B*[1], cultured human cells contain a novel *UEV1* splicing variant, *UEV1C*, lacking the N-terminal 30 amino acid unique region of Uev1A. The resulting 147-residue protein would co-migrate with Mms2 during electrophoresis. It turns out that the *UEV1C* transcript is much more abundant than *UEV1A*, and a Uev1-specific monoclonal antibody can detect cellular Uev1C but not Uev1A, unless the latter is experimentally overexpressed.

The current study investigates roles of *UEV1A*, *UEV1C* and *MMS2* in tumorigenesis using a breast cancer model. With comparable levels of ectopic expression, it was found that only *UEV1A*, but not *UEV1C* or *MMS2*, is able to promote cell migration and invasion. Similarly, overexpression of *UEV1A*, but not *UEV1C* promotes tumor growth and metastasis in a xenograft mouse model. The above results are highly reliable, as the target gene expression is under tight regulation of a Tet-on promoter, and the phenotypes were only observed under Dox-induced conditions. In a reverse experiment, depletion of Uev1 in cultured breast cancer cells significantly reduces cell migration and invasion, as well as tumor growth and metastasis in a dose-dependent manner, indicating that the cellular Uev1 (presumbly Uev1A) level plays a critical role in breast tumorigenesis and metastasis.

To understand the molecular mechanism by which Uev1A promotes tumorigenesis, we demonstrated that overexpression of *UEV1A*, but not *UEV1C* or *MMS2*, is able to promote IκBα phorsphorylation and NF-κB translocation into the nucleus, and that this effect absolutely relies on its physical interaction with Ubc13. It is conceivable that as previously reported, the Ubc13-Uev1A heteromider serves as an E2 to assemble K63-linked poly-Ub chains along with cognate really interesting new gene (RING)-finger E3s like TRAF2 and/or TRAF6 [17,34], which recruit K63 polyUb-binding proteins like NEMO [18] and TAB2/3 [47,48] to phorsphorylate and subsequently degrade IκBα, leading to NF-κB activation.

NF-κB activation promotes the transcription of many downstream genes in the signaling cascade [41]. However, the moderate level of NF-κB activation by *UEV1A* overexpression does not appear to induce all NF-κB targeting genes. To understand how overexpression of *UEV1A* leads to tumorigenesis and particularly metastasis in breast cancer cells, we surveyed NF-κB and metastasis-related genes and focused on two candidate genes, *MMP1* and *MMP9*, both of which are highly induced upon *UEV1A* overexpression. Experimental results as presented in this report indicate that both genes are tightly regulatd by cellular Uev1 levels; however, depletion of MMP9 was not as effective as that of MMP1 on cell migration and invasion. As ecotopic expression of *MMP1* to restore the wild-type level in Uev1-depleted cells also restored wild-type level of invasiveness, it is plausible to conclude that *MMP1* is the critical downstream effector of *UEV1A*-induced breast cancer metastasis, although this study does not rule out the contributions of *MMP9* and possibly other genes. The signal transduction cascade of Uev1A → NF-κB → MMP1 → metastasis is further confirmed by showing that Uev1A-induced *MMP1* expression is dependent on both Ubc13 and the predicted NF-κB binding site located in the *MMP1* promoter. Although this report only presents data from one human breast cancer line, we have obtained comparable results with a different breast cancer cell line MCF7 (data not shown), indicating that the tumorigenic and metastatic effects of Uev1A is a general phenomenon in breast cancers.

While the experimental evidence as shown in this report clearly indicates that *UEV1A* can function as a proto-oncogene, the clinical relevance of this finding awaits future investigation. Nevertheless, our limited TissueScan microarray data indicate a low (less than 2-fold) variation in *UEV1A* transcript levels among five normal human breast samples, compared with an increase of up to 20-fold in some breast cancer samples. As NF-κB activation is commonly observed in breast cancers [24,25,49] and *UEV1* upregulation is also frequently observed in breast cancer samples [12-14] and in cultured tumor cell lines [4], and is found to be correlated to tumorigenic indicators [2,3], it is conceivable that a certain percentage of breast cancer samples with NF-κB activation is due to elevated *UEV1A* expression.

This study demonstrated that the N-terminal region of Uev1A is the molecular determinant of its cellular function(s) in the NF-κB signaling pathway. Although the exact cellular function of Uev1C remains a mystery, our previous studies [10] have shown that truncated Uev1A missing the N-terminal 30 amino acids behaves like Mms2 in terms of subcellular localization and promotion of K63-linked di-Ub versus poly-Ub chains *in vitro*, suggesting that Uev1C may play an Mms2-related role.

Given the importance of Uev1 in signaling and tumorigenesis, small-molecule inhibitors against Uev1 have been isolated [20,50] based on their interference with the Ubc13-Uev1A interaction, and one appears to be able to inhibit proliferation and survival of diffuse large B-cell lymphoma cells. It is unclear whether these inhibitors also interfere with the Ubc13-Mms2 interaction, as critical residues responaible for the heterdimer formation are conserved between Uev1 and Mms2 [9]. Furthermore, this study provides evidence that a desired inhibitor should target the N-terminal region of Uev1A instead of the Ubc13-Uev1 interface. Hence, this report provides an experimental and theoretical cornerstone for future diagnosis and therapy by targeting Uev1A for the cure of breast cancer.

## Conclusions

Among three Uev gene products, only Uev1A promotes breast tumor metastasis, which is primarily through activating the NF-κB target gene *MMP1*. Hence, *UEV1A* is considered a proto-oncogene and a therapeutic target for breast cancers.

## Abbreviations

DMEM: Dulbecco's modified Eagle's medium; Dox: doxycycline; EGF: epidermal growth factor; EMSA: electrophoretic mobility shift assay; H&E: hematoxylin and eosin; HRP: horseradish peroxidase; IL: interleukin; IκBα: inhibitor of NF-κB alpha; MMP1: matrix metalloproteinase-1; NF-κB: nuclear factor of kappa-light polypeptide gene enhancer in B-cells; PBS: phosphate-buffered saline; PCR: polymerase chain reaction; shRNA: small hairpin RNA; siRNA: small interfering RNA; TNF: tumor necrosis factor; TRAF: tumor necrosis factor receptor-associated factor; UEV: ubiquitin conjugating enzyme variant.

## Competing interests

The authors declare they have no competing interests.

## Authors’ contributions

ZW participated in the project design and carried out most experiments. SS carried out the *MMP1* expression and restoration experiments in MDA-MB-231 cells and cell mobility analysis in MCF7 cells. ZZ carried out MMP1 and MMP9 depletion by siRNA and analysis in MDA-MB-231 cells, HeLa cell nuclear protein extraction, and participated in the animal studies. WZ was involved in project design and technical advice. WX conceived the study, participated in the project design, manuscript preparation and submission. All authors read and approved the final manuscript.

## Supplementary Material

Additional file 1 Clinical data for the TissueFocus™ breast cancer tissue microarray purchased from Origene. Click here for file

Additional file 2 **Figure S1. ***Ubiquitin conjugating enzyme variant* (*UEV*)*1A* is overexpressed in breast cancer cell lines and tumor samples. **Figure S2.***UEV* expression levels in MDA-MB-231-TR inducible cells. **Figure S3. ***UEV* overexpression does not affect cell cycle progression or proliferation in MDA-MB-231 cells. **Figure S4.** Representative images of wound-healing assays without doxycycline (Dox) treatment. **Figure S5.** Uev1 depletion reduces cell invasion *in vitro* and tumor growth in a xenograft model. **Figure S6. ***Matrix metalloproteinase* (*MMP*)*1* is tightly regulated by *UEV1*. Click here for file
