# Erratum to: Ubiquitin-conjugating enzyme complex Uev1A-Ubc13 promotes breast cancer metastasis through nuclear factor-кB mediated matrix metalloproteinase-1 gene regulation

**DOI:** 10.1186/s13058-017-0833-6

**Published:** 2017-03-28

**Authors:** Zhaojia Wu, Siqi Shen, Zhiling Zhang, Weiwei Zhang, Wei Xiao

**Affiliations:** 1 College of Life Sciences, Capital Normal University, Beijing, 100048 China; 2 Department of Microbiology and Immunology, University of Saskatchewan, Saskatoon, S7N 5E5 Canada

## Erratum

After the publication of this work [1] an error was noticed in Fig. 2d, in which an image from *UEV1C* transfected cells without Dox treatment (Dox^-^) was mistakenly presented as *MMS2* transfected Dox- cell image. The corrected Fig. 2 that contains a replacement image for *MMS2* transfected Dox^-^ cells in Fig. 2d is presented. As *UEV1C* and *MMS2* transfections have indistinguishable effects on cell invasion (Fig. 2e), the correction does not affect our conclusions. Nevertheless, we apologize for this error.

**Fig. 2:**
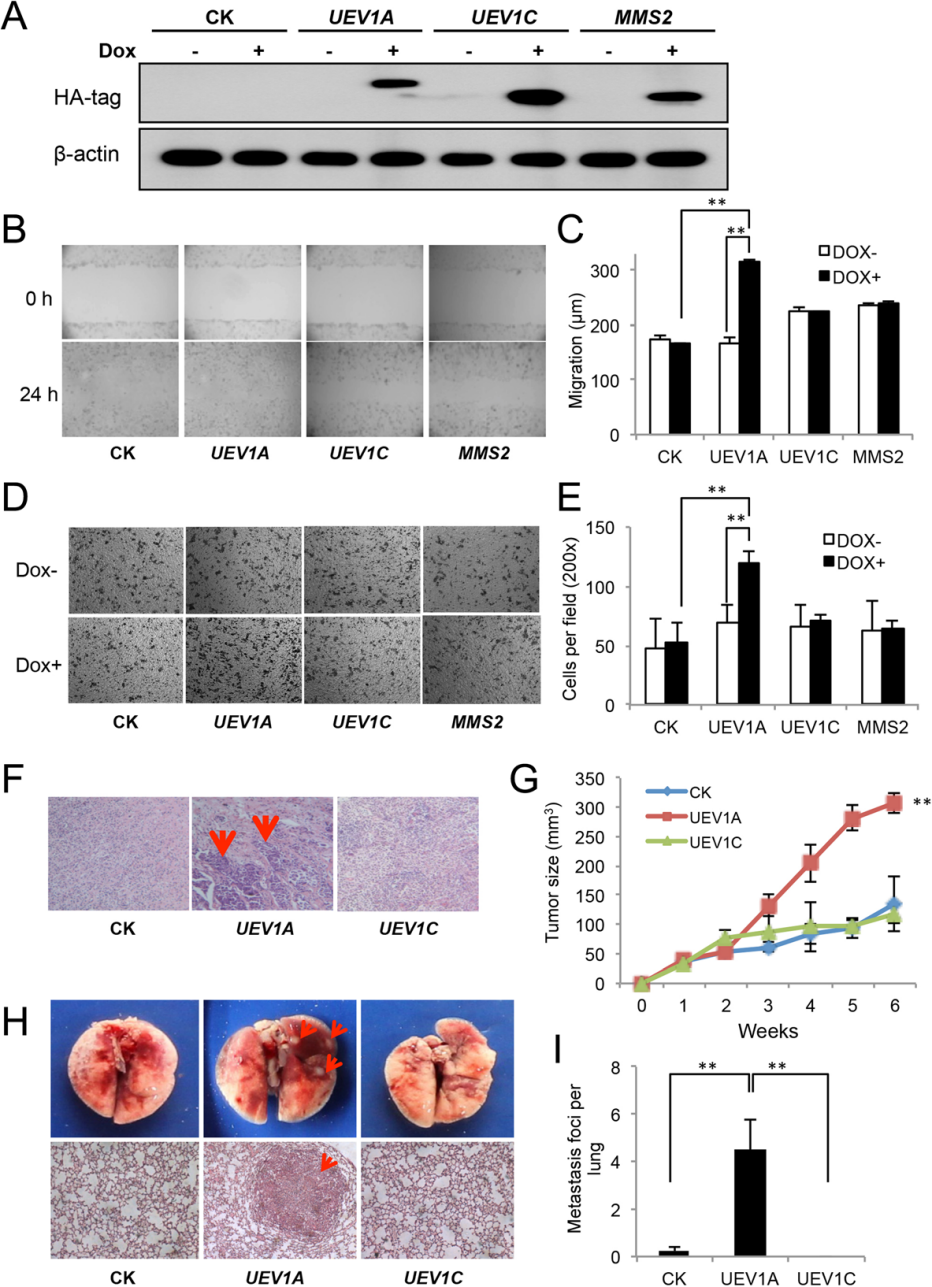
*UEV 1A* overexpression promotes breast cancer cell invasion *in vitro* and metastasis in a xenograft model. **a** pcDNA4.0/TO/HA(+) vector (CK) expressing *UEV1A*, *UEV1C* or *MMS2* was stably transfected into MDA-MB-231-TR cells, with or without doxycycline (Dox) treatment. The level of ectopic gene expression was monitored by western blot against an anti-HA antibody. **b** Representative images of wound-healing assays with Dox treatment. **c** Statistical analysis of cell migration of wound-healing assay with and without Dox treatment. The migration distance of cells was measured in five different wells in each group under a light-microscope. **d** Representative images of cell invasion assay with Matrigel-coated transwells. **e** Statistical analysis of the cell invasion assay data. Cells that invaded the lower surface of the filter were counted in five random fields under a light-microscope at 200× magnification. **f**-**i** *In vivo* tumorigenesis and metastasis assays using a xenograft mouse model. **f** Lymph node sections after sacrifice were stained with H&E. The lymph node metastasis sites are shown by red arrows. **g** Quantitative analysis of tumor growth. Tumor growth was measured every week after injection (Day 0) and expressed as mean ± SD (n = 10). **h** The *in vivo* metastasis assay in xenograft mice. Upper panel, the lung metastasis nodules formed are shown by red arrows. Lower panel, the lung sections were stained with H&E and the lung metastasis under a light-microscope at 100× magnification is indicated by a red arrow. **i** Quantitative analysis of the *in vivo* lung metastasis as measured by the number of metastasis foci per lung for all four sections (n = 10 mice for each treatment)

## References

[1] Wu Z, Shen S, Zhang Z, Zhang W, Xiao W (2014). Ubiquitin-conjugating enzyme complex Uev1A-Ubc13 promotes breast cancer metastasis through nuclear factor-small ka, CyrillicB mediated matrix metalloproteinase-1 gene regulation. Breast Cancer Res.

