# Supplementary material for: Ubiquitin-conjugating enzyme complex Uev1A-Ubc13 promotes breast cancer metastasis through nuclear factor-кB mediated matrix metalloproteinase-1 gene regulation
Source: Breast Cancer Res. 2014 Jul 14;16(4):R75. doi: 10.1186/bcr3692 (PMC4223564; doi:10.1186/bcr3692)
Supplement: Additional file 2 — Figure S1. Ubiquitin conjugating enzyme variant (UEV)1A is overexpressed in breast cancer cell lines and tumor samples. Figure S2.UEV expression levels in MDA-MB-231-TR inducible cells. Figure S3. UEV overexpression does not affect cell cycle progression or proliferation in MDA-MB-231 cells. Figure S4. Representative images of wound-healing assays without doxycycline (Dox) treatment. Figure S5. Uev1 depletion reduces cell invasion in vitro and tumor growth in a xenograft model. Figure S6. Matrix metalloproteinase (MMP)1 is tightly regulated by UEV1. [file bcr3692-S2.doc]

**Additional file 2**

**Figure S1** ***UEV1A* is overexpressed in breast cancer cell lines and tumor samples.** **(A)** Relative *MMS2* transcript levels in human breast cancer cell lines as determined by qRT-PCR. **(B)** Relative *UEV1A* transcript levels in a TissueScan microarray as determined by qRT-PCR. The first five samples are from normal human breast tissues, while the remaining 43 are breast cancer samples. The blue horizontal line indicates the average *UEV1A* level in the five normal samples, which is set at 1, while the red horizontal line marks the highest *UEV1A* level among the five normal samples. The clinical information for the above samples can be found on the website http://www.origene.com/qPCR/Tissue-qPCR-Arrays.aspx.

**Figure S2** ***UEV* expression levels in MDA-MB-231-TR inducible cells.** **(A)** Relative *UEV1A* transcript levels in MDA-MB-231-TR cells with or without Dox induction as determined by qRT-PCR. **(B)** Relative *UEV1C* transcript levels in MDA-MB-231-TR cells with or without Dox induction as determined by qRT-PCR. **(C)** Relative *MMS2* transcript levels in MDA-MB-231-TR cells with or without Dox induction as determined by qRT-PCR. **(D)** Endogenous and ectopic Uev1A and Uev1C protein levels in MDA-MB-231-TR cells were determined by western blot against a Uev1-specific mAb LN2B. **(E)** Endogenous Mms2/Uev1 and ectopic Mms2 protein levels in MDA-MB-231-TR cells were determined by western blot against the pan-Mms2/Uev1 mAb LN3.  **(F)** Endogenous Uev1 and ectopic Uev1A-F38E protein levels in MDA-MB-231-TR cells were determined by western blot against LN2B. Western blots of β-actin are used as internal controls.

**Figure S3** ***UEV* overexpression does not affect cell cycle progression or proliferation in MDA-MB-231 cells.** (**A**) MDA-MB-231-TR cells expressing ectopic *UEV* genes were harvested and stained with DAPI for cell-cycle distribution analysis. The percentage of cells in different cell cycle stages is indicated. (**B**) Growth curve of MDA-MB-231-TR cells expressing ectopic *UEV* genes. Viable cells were counted at the given time intervals and expressed as number of cells per view. The cells carrying an empty vector served as control. Results are the average of three independent experiments with standard deviations.

**Figure S4 Representative images of wound-healing assays without Dox treatment.** Experiment conditions are the same as described in Figure 2B, but without Dox treatment.

**Figure S5** **Uev1 depletion reduces cell invasion *in vitro* and tumor growth in a xenograft model**. (**A-E)**. MDA-MB-231 cells were transfected with shRNA lentiviral particles either against *UEV1* (sh*UEV1*) or non-specific target (shCK). The transcript levels of (**A**) *UEV1C* and (**B**) *MMS2* in two independent shRNA lines were determined by qRT-PCR. (**C**) Uev1 (LN2B) and Mms2+Uev1 (LN3) protein levels in two independent shRNA lines were determined by western blot. (**D**) Cell migration was determined by the wound-healing assay. (**E**) Cell invasion was determined by the Matrigel-coated transwell assay. (**F**) Tumor formation in nude mice at the fifth week after injection of treated MDA-MB-231 cells into lateral ﬂanks. Tumors are marked by red brackets. Quantitative analyses of images shown in (D-F) are given in Figure 3B-D.

**Figure S6 *MMP1* is tightly regulated by *UEV1***. (**A**) Representative images showing that depletion of MMP1 or MMP9 limits MDA-MB-231 cell invasion. After MMP1 or MMP9 depletion for 48 hrs, MDA-MB-231 cells were subject to an invasion assay in Matrigel-coated transwells as previously described. (**B**) The relative transcript levels of *MMP1*, *MMP9* and *UEV1A* in *UEV1A*-overexpressed MDA-MB-231 cells after MMP1 or MMP9 depletion, as determined by qRT-PCR. (**C**) *UEV1A*-overexpressed MDA-MB-231 cells depleted with MMP1 or MMP9 were subject to the transwell assay and at least 5 random ﬁelds were counted under a light microscope at 200x magniﬁcation. (**D**) Representative images showing that depletion of MMP1 or MMP9 reverses the effects of *UEV1A* overexpression on cell invasion. (**E**) The relative *MMP1* transcript levels in control and *MMP1*-expressing MDA-MB-231 cells as determined by qRT-PCR. (**F**) MMP1 protein levels in the control and MMP1-expressing MDA-MB-231 cells as determined by western blot. β-actin was used as a loading control. (**G**) Representative images showing that *MMP1* overexpression stimulates cell invasion in a Matrigel-coated transwell assay.


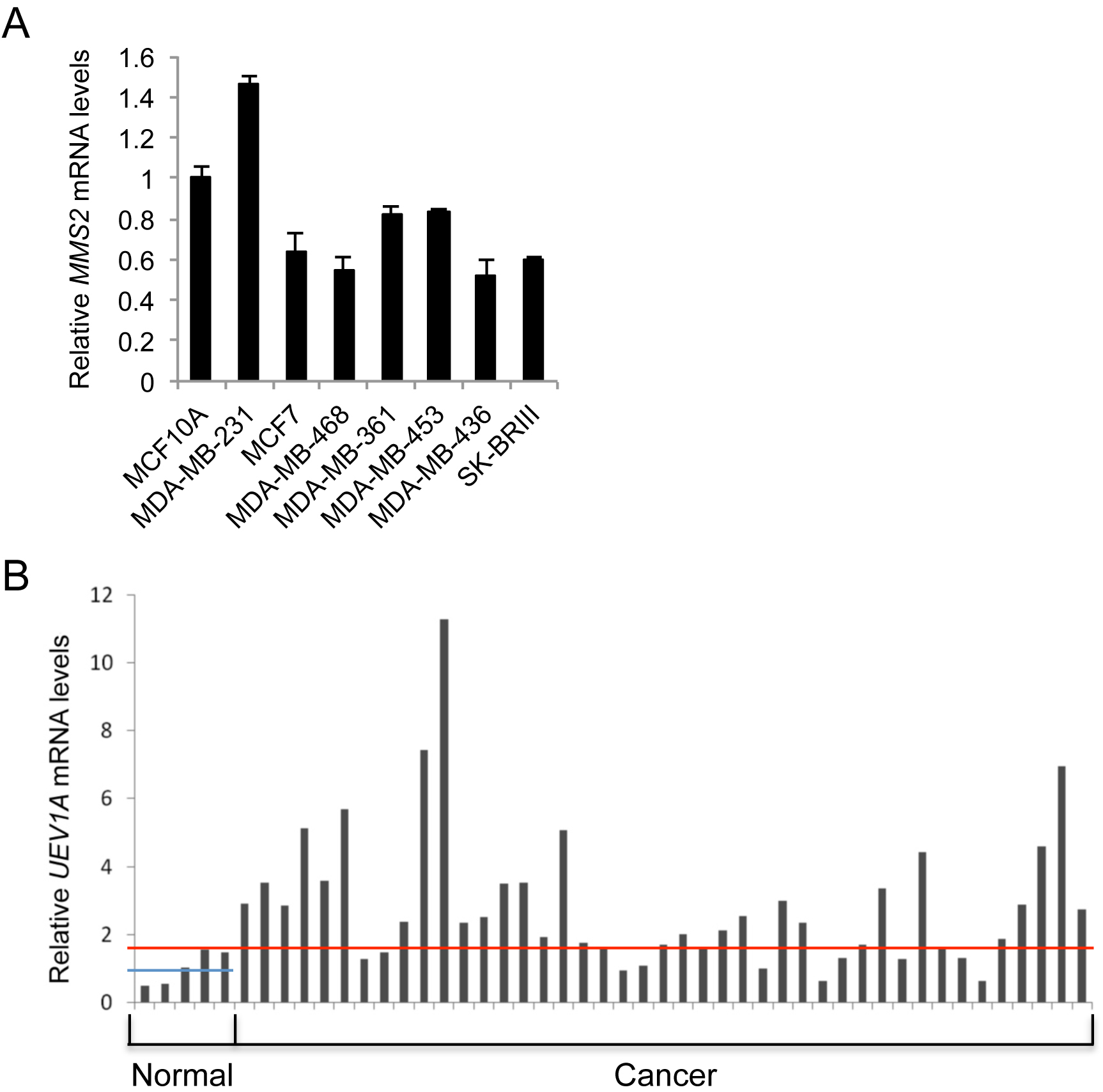


**Figure S1**


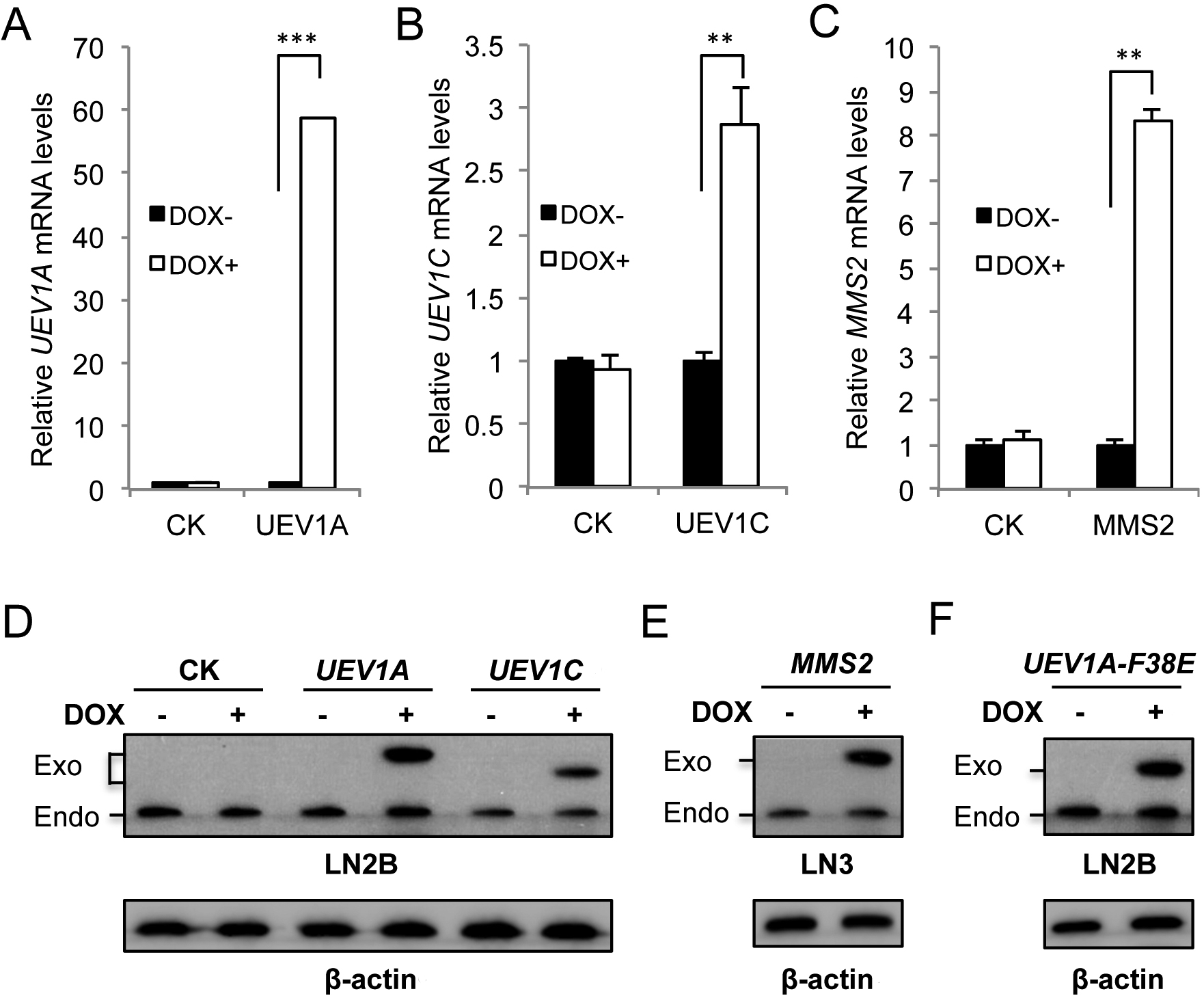


**Figure S2**


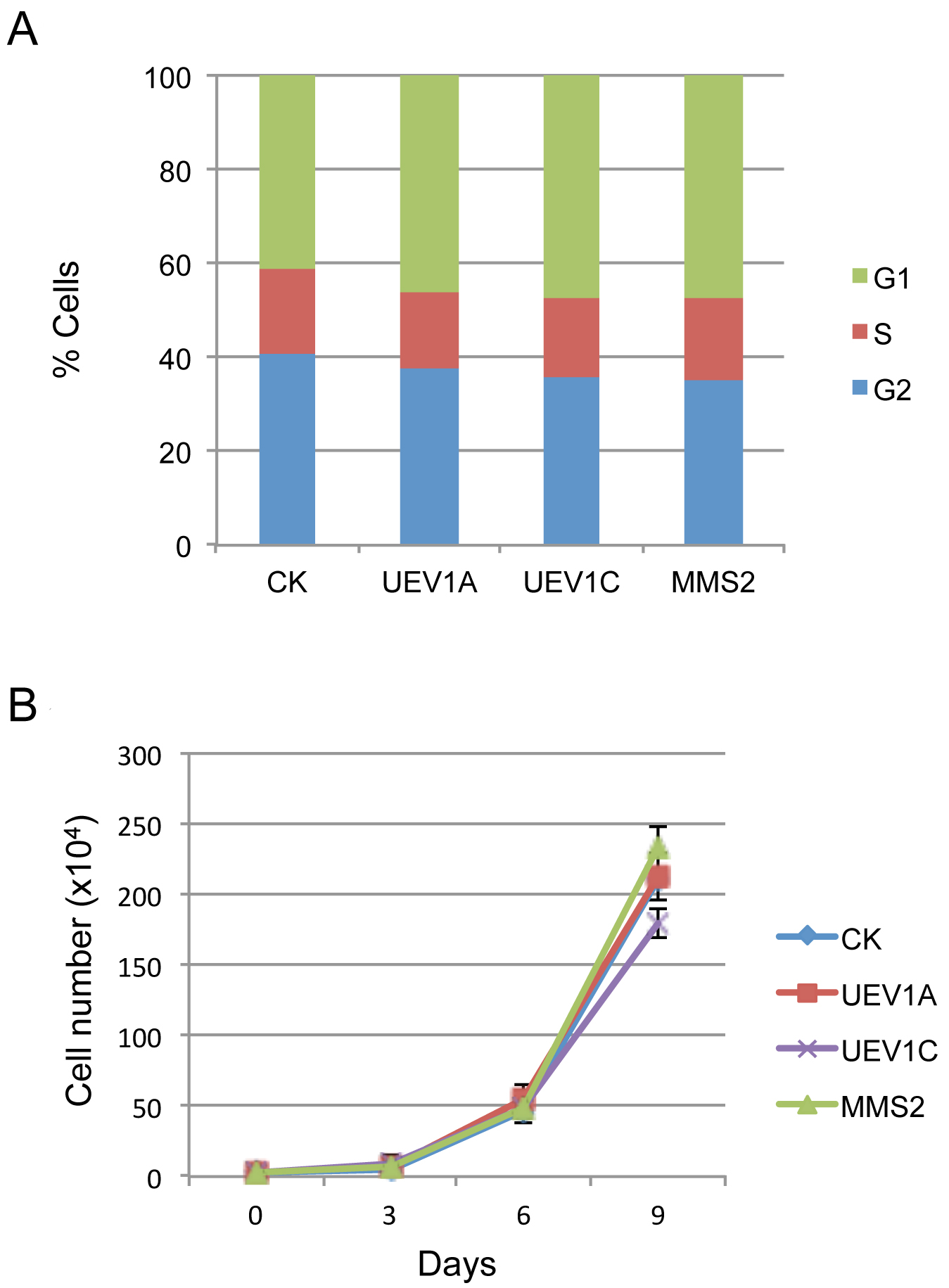


**Figure S3**


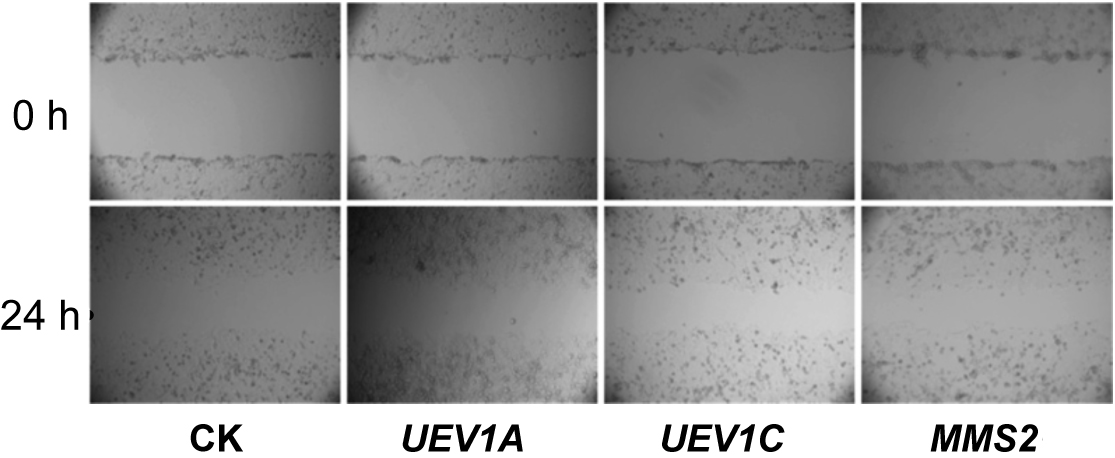


**Figure S4**


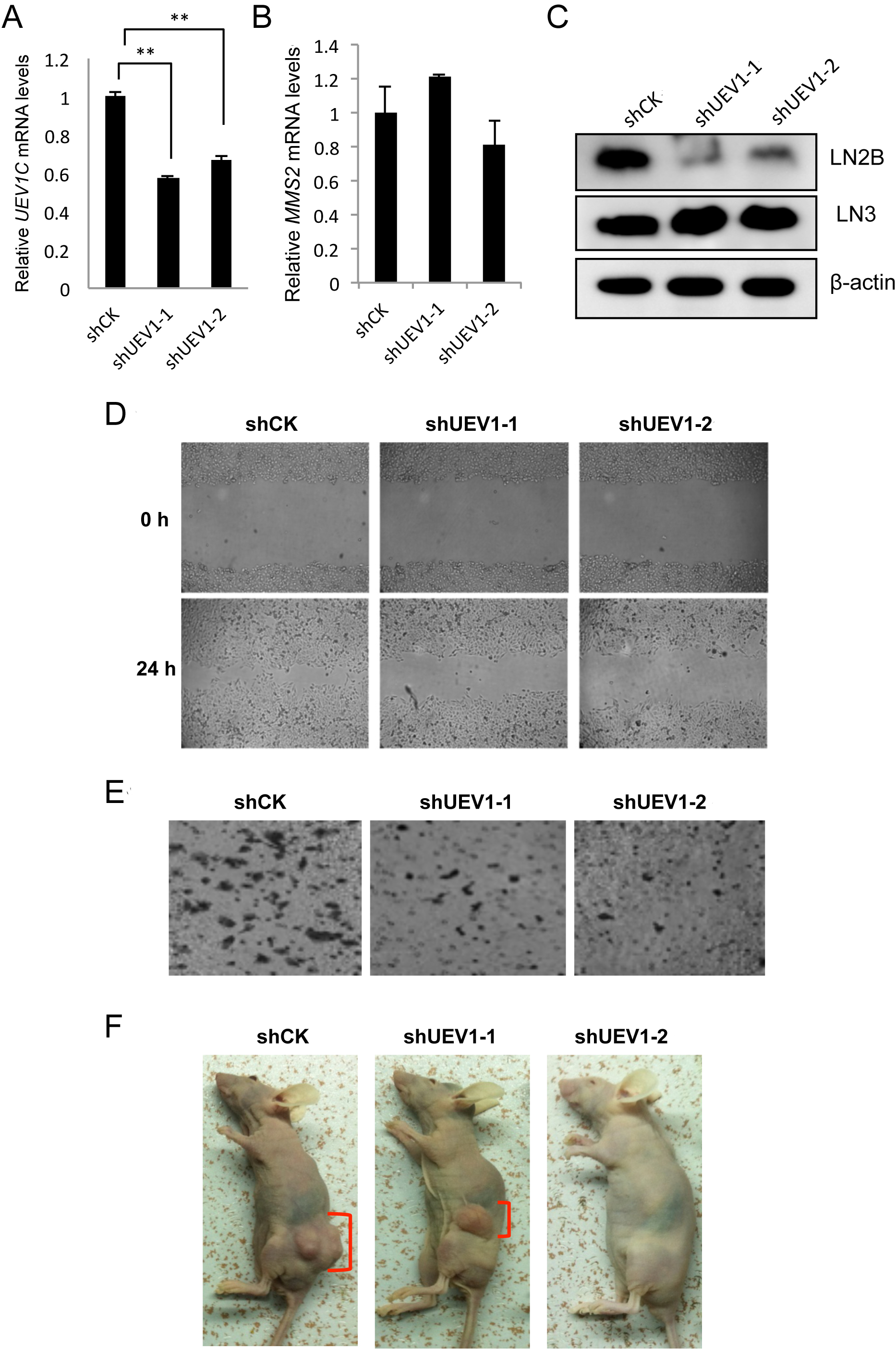


**Figure S5**


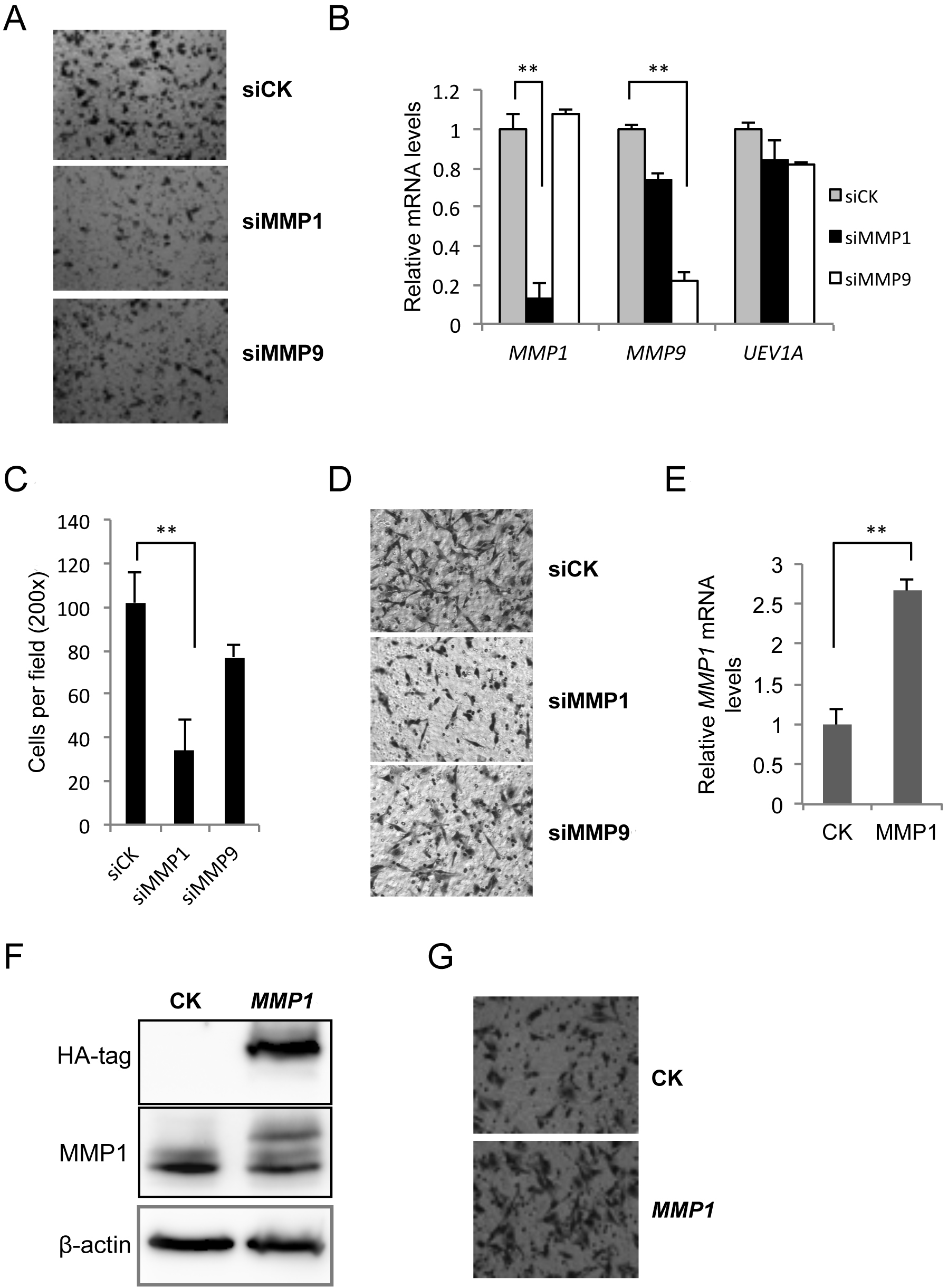


**Figure S6**
